# 3D structures inferred from cDNA clones identify the CD1D-Restricted γδ T cell receptor in dromedaries

**DOI:** 10.3389/fimmu.2022.928860

**Published:** 2022-08-09

**Authors:** Giovanna Linguiti, Vincenzo Tragni, Ciro Leonardo Pierri, Serafina Massari, Marie-Paule Lefranc, Rachele Antonacci, Salvatrice Ciccarese

**Affiliations:** ^1^ Department of Biology, University of Bari “Aldo Moro”, Bari, Italy; ^2^ Department of Biosciences, Biotechnologies and Biopharmaceutics, University of Bari “Aldo Moro”, Bari, Italy; ^3^ Department of Biological and Environmental Science and Technologies, University of Salento, Lecce, Italy; ^4^ The International ImMunoGeneTics Information System^®^ (IMGT^®^), Laboratoire d’ImmunoGénétique Moléculaire (LIGM), Institut de Génétique Humaine (IGH), Montpellier, France

**Keywords:** T cell receptor, *Camelus dromedarius*, TRG and TRD loci, multiple sequence alignments (MSA), 3D modelization, IMGT unique numbering

## Abstract

The Camelidae species occupy an important immunological niche within the humoral as well as cell mediated immune response. Although recent studies have highlighted that the somatic hypermutation (SHM) shapes the T cell receptor gamma (TRG) and delta (TRD) repertoire in *Camelus dromedarius*, it is still unclear how γδ T cells use the TRG/TRD receptors and their respective variable V-GAMMA and V-DELTA domains to recognize antigen in an antibody-like fashion. Here we report about 3D structural analyses of the human and dromedary γδ T cell receptor. First, we have estimated the interaction energies at the interface within the human crystallized paired TRG/TRD chains and quantified interaction energies within the same human TRG/TRD chains in complex with the CD1D, an RPI-MH1-LIKE antigen presenting glycoprotein. Then, we used the human TRG/TRD-CD1D complex as template for the 3D structure of the dromedary TRG/TRD-CD1D complex and for guiding the 3D human/dromedary comparative analysis. The choice of mutated TRG alternatively combined with mutated TRD cDNA clones originating from the spleen of one single dromedary was crucial to quantify the strength of the interactions at the protein-protein interface between the paired *C. dromedarius* TRG and TRD V-domains and between the *C. dromedarius* TRG/TRD V-domains and CD1D G-domains. Interacting amino acids located in the V-domain Complementarity Determining Regions (CDR) and Framework Regions (FR) according to the IMGT unique numbering for V-domains were identified. The resulting 3D dromedary TRG V-GAMMA combined with TRD V-DELTA protein complexes allowed to deduce the most stable gamma/delta chains pairings and to propose a candidate CD1D-restricted γδ T cell receptor complex.

## Introduction

T cell populations are characterized by two lymphocyte subsets that express distinct heterodimeric antigen specific receptors (TRs) formed by alpha and beta chains (αβ T cells) or by gamma and delta chains (γδ T cells). γδ T cells have unique features compared to the more abundant αβ T cells, e.g., a preferential distribution in both epithelial and mucosal sites, and an immunoglobulin like antigen recognition mechanism. In the immune response during inflammatory processes, γδ T cells release cytokines and kill infected macrophages. Despite decades of research, we still have little information regarding how many defined γδ T cells populations exist, what antigens/ligands they respond to and what roles they play in host defense. A diverse population of human γδ T cells that exhibit CD1D autoreactivity has recently been identified and characterized (1). Natural Killer T (NKT) cells are CD1D-restricted innate-like T cells expressing both T cell receptor and NK cell markers. The major group of NKT cells in both human and mice is the invariant NKT (iNKT) cells and the best-known function of iNKT cells is their potent anti-tumor function in mice (2). In camelids that occupy an important immunological niche within the humoral as well as cell mediated immune response, it is still unclear how γδ T cells use their V-(D)-J rearranged T cell receptor (TR) gamma/delta heterodimers to recognize antigen in an antibody-like fashion (3). In previous works we provided evidence that TR gamma variable (TRGV) and delta variable (TRDV) productively rearranged genes expressed in *Camelus dromedarius* spleen undergo somatic hypermutation (SHM) (4). Conversely, in ab T cells, the limited TR beta variable (TRBV) germline repertoire in *C. dromedarius* is not shaped by SHM and it might be related to the constraint imposed on the CDR1-IMGT and CDR2-IMGT (5) domains of the αβ V-domains by the requirements for binding to MH molecules (6). In this report, based on the progresses of immunoinformatic (7), we used a computational approach for further analyzing at molecular level the human TR gamma (TRG) and delta (TRD) chains crystallized in complex with the antigen presenting glycoprotein CD1D, a RPI MH1-LIKE protein associated to beta-2-microglobulin (B2M) (4LHU) (1, 8) highlighted along pGenThreader (http://bioinf.cs.ucl.ac.uk/psipred/) and I-tasser (https://zhanglab.ccmb.med.umich.edu/I-TASSER/) searches (8), while searching for the ideal protein template to model the dromedary TRG and TRD chains. Thus, we quantified the interaction energies at the TRG/TRD protein-protein interface between the V-domains of the human structure. The human TR (4LHU) structure was the ideal protein template for guiding the 3D human/dromedary comparative modeling analysis. Starting from the cDNA sequences of dromedary mutated clones originating from the spleen of one single subject, we were able to build a 3D model for each dromedary TRG/TRD pair and to deduce the most efficient pairing of the TRG and TRD chains. In addition, given that the human TRG/TRD was crystallized in complex with CD1D and B2M, it was possible to build a 3D model of the dromedary TR gamma/delta in complex with the dromedary CD1D and B2M. Finally, we estimated the resulting interaction energies at the TRG/TRD-CD1D protein-protein interface within the generated dromedary 3D models. Furthermore, it was possible to highlight the interacting amino acids in the CDR-IMGT (5) (IMGT^®^ http://www.imgt.org) of the generated dromedary TR/CD1D structure.

## Methods

### Crystal structure sampling *via* folding recognition and multiple sequence alignments

The folding recognition methods implemented in pGenThreader (http://bioinf.cs.ucl.ac.uk/psipred/) and I-Tasser (https://zhanglab.ccmb.med.umich.edu/I-TASSER/) were used for identifying TR gamma (TRG) and TR delta (TRD) homologous protein-crystallized structures to be used as a protein template for generating 3D all atom comparative models of our investigated dromedary gd T cell receptors. With this aim the amino acid (AA) sequence of the *Camelus dromedarius* TRG (AFD98894.1 and AFD98918.1; accession JF755952.1 and JF792640.1) and TRD proteins (CAX52224.1, CAX52229.1 and CAX52219.81; accession FN252371.1, FN252376.1 and FN252345) were used as query sequences for running pGenThreader and I-Tasser to screen the PDB, searching for the most similar deposited crystallized structures (9–12). Swiss PDBV Viewer (SPDBV: https://spdbv.unil.ch/) was used for building a 3D all atom model of all the investigated *C. dromedarius* TRG or TRD chains by using the *Homo sapiens* TRG or TRD chains available in the human crystallized TR γδ in complex with antigen presenting glycoprotein CD1D, a RPI MH1-Like protein associated to beta-2-microglobulin (B2M) (1), available under the pdb code “4LHU” in the RCSB Protein Data Bank (PDB) and in IMGT/3Dstructure-DB (13). The *Homo sapiens* CD1D and B2M sequences were used as query sequences for identifying their counterparts in *C. dromedarius*, through blastp searches. The identified protein sequences were modelled by using the structures of the *Homo sapiens* CD1D and B2M available under the pdb code “4LHU”, used as a protein template, by SPDBV according to our validated protocols (8, 12, 14). All the generated 3D all-atom models were energetically minimized by using the Yasara Minimization server (8, 14, 15).

### 3D modeling of paired *C. dromedarius* TRG and TRD V-domains in complex with *C. dromedarius* CD1D G-domains and B2M

The proposed 3D comparative protein complex consisting of paired *C. dromedarius* TRG and TRD V-domains in complex with *C. dromedarius* CD1D G-domains and B2M was obtained by superimposing the *C. dromedarius* V-domains of TRG (RTS88 and 5R1S169) and TRD (RTVD4m9, RTVD4m14 and JD3.05) clones on the corresponding 3D atomic coordinates of each corresponding chain within the crystallized human TR γδ in complex with the *Homo sapiens* CD1D and B2M, available under the PDB code LHU (16), by PyMOL as previously described (8, 14) Superimposition operations were performed through the “super” command implemented in PyMOL, starting from the structural alignment of the alpha Carbons (Cα) of both protein models (17). The “super” command allows aligning the selected proteins under investigation for performing a comparative structural analysis, due to its ability in providing a sequence-independent structure-based pairwise alignment. Notably, the “super” command is more robust than the “align” command because it successfully performs also superimposition of proteins with a lower sequence similarity (8, 11, 14, 17). Then, it was possible to model/relax missing/buried residues located at the protein-protein interface, solving clashes and putative breaks in the backbone (11, 12, 14, 17). All the generated 3D all-atom models were energetically minimized by using the Yasara Minimization server (8, 14, 15). The obtained final models were examined in VMD (https://www.ks.uiuc.edu/Research/vmd/), PyMOL (https://pymol.org/2/), and SPDBV by visual inspection searching for putative unsolved clashes (8, 12, 14). Protein-protein binding regions were highlighted by selecting amino acid residues within 4 Å at the protein-protein interface, in the superimposed structures.

### FoldX energy calculations

**Figure 1:**
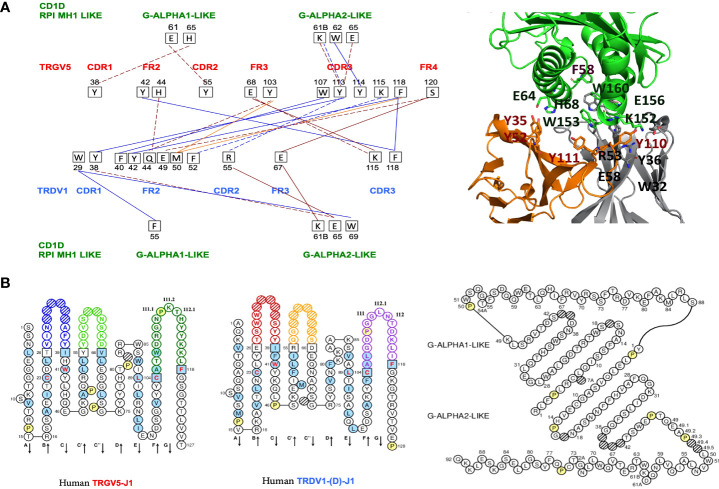
**(A)** Protein-protein interactions between the Homo sapiens FR-IMGT and CDR-IMGT of the TR V-GAMMA and V-DELTA domains and between the TR V-GAMMA or V-DELTA domains and the Homo sapiens CD1D G-ALPHA1-LIKE and G-ALPHA2-LIKE domains. Five types of interactions are shown: protein-protein ionic interactions within 6 Å (solid brown line), protein-protein side chain-side chain hydrogen bonds (broken brown line), aromatic-aromatic interactions within 4.5 and 7- Å (solid blue line), aromatic-sulphur interactions (solid red line), cation-Pi interactions (broken blue line) (11, 12, 14),. On the left, in red the CDR and FR of the TRGV5 V-GAMMA with the respective amino acids and their positions involved in the interactions (black squares). In blue the CDR and FR of the TRDV1 V-DELTA. In green the CD1D G-ALPHA1-LIKE and the G-ALPHA2-LIKE domains, with their respective amino acids and their positions involved in the interactions with the V-GAMMA and V-DELTA domains. In the upper right, interacting amino acids at the interface of the cited TR gd 3D models with the Homo sapiens CD1D G-ALPHA1-LIKE, and G-ALPHA2-LIKE domains are reported in sticks representation (See also **Figure 2B** and **Table S2**). **(B)** IMGT Collier de Perles of the Homo sapiens TRGV5-TRGJ1 domain, the TRDV1-(D)-J1 domain (18, 19) and the G-ALPHA1-LIKE and the G-ALPHA2-LIKE domains of the CD1D, available in IMGT/3Dstructure-DB, http://www.imgt.org, accessed on 4 March 2022.

The FoldX Analyze Complex assay was performed to determine the interaction energy between the paired *C. dromedarius* TRG and TRD V-domains (V-GAMMA and V-DELTA) and between the paired TRG/TRD V-domains in complex with the *C. dromedarius* CD1D and B2M, within the six 3D modelled protein complexes (2 TRG V-domains alternatively combined with 3 TRD V-domains) but also for determining the interaction energy between the *Homo sapiens* counterparts of the crystallized 4lhu.pdb as a reference system, as validation strategy and for comparative purposes (**Table S1**; **Figure S1** **).** We have converted the amino acid positions listed in **Table S1** to the corresponding positions in the IMGT unique numbering for both the V-domains (18) and the G-domains (19). For the V-domains, this allows to highlight the complementarity determining regions (CDR), in contact with the antigen, and the framework regions (FR) of the V-domains, and to represent the TRGV5-J1 and TRDV1-(D)-J1 domains of “4lhu.pdb” with standardized CDR-IMGT and FR-IMGT, using the IMGT/Collier-de-Perles tool for V-domains. For the G-domains, this allows to identify the positions of the helices and of the groove floor of the CD1D G-domains (G-ALPHA1-LIKE and G-ALPHA2-LIKE) and to represent the two G-domains, using the Analyze IMGT/Collier-de-Perles tool for G-domains (20) (**Figure S1**, **Figures 1A, B**; **Table S2** **).**

The way the FoldX AnalyseComplex operates is by unfolding the selected targets and determining the stability of the remaining molecules and then subtracting the sum of the individual energies from global energy. More negative energies indicate a better binding. Positive energies indicate no binding (**Table S3** **)** (21).

## Results

### Analysis of the human CD1D-restricted γδ T cell receptor

The crystal structure with PDB code 4LHU consisting of the *Homo sapiens* TR gamma-delta in complex with the antigen presenting glycoprotein CD1D, a RPI MH1-LIKE protein associated to beta-2-microglobulin (B2M), was identified along pGenThreader and I-tasser searches as the ideal protein template for guiding the 3D comparative modeling analysis. The identified protein sequences were modelled by using the structures of the *Homo sapiens* CD1D and B2M available in RCSB PDB and in IMGT/3Dstructure-DB (13) under the pdb code “4lhu.pdb”, used as a protein template (1).

To validate our protocol, we first quantify the TRG and TRD chains pairing interactions with each other through the amino acid interactions at the interface between their V-domains, V-GAMMA and V-DELTA. We then quantify the type of interactions at the interface between each V-domain and each G-domain of CD1D (**Figure 1**; **Figure S1**).

We identified and analyzed six types of Interactions: protein-protein ionic interactions within 6 *Å*, protein-protein side chain-side chain hydrogen bonds, aromatic-aromatic interactions within 4.5 and 7 Å, aromatic-sulphur interactions, cation-Pi interactions, hydrophobic interactions within 5 Å (**Table S1**; **Figure S1**). Five types of interactions are shown in **Figures 1A, B** which identify the interacting amino acids at the protein interface between the TRG and TRD V domain pairing. It is possible to note how the amino acids of the V-DELTA FR2-IMGT interact with the amino acids of the V-GAMMA CDR3-IMGT. Moreover, TRD V-domain CDR1 (germline encoded) is involved in the interactions with CD1D G-ALPHA 1-LIKE by aromatic-aromatic (W29-F55) and with G-ALPHA 2-LIKE by aromatic-aromatic (W29-W69) and side chain-side chain hydrogen bonds (Y38-E65). The same type of interactions together with the Cation-π interaction (Y113-K61B) was found between the TRG V-domain CDR3 and the CD1D G-ALPHA 2 **(** **Figures 1A, B** **)**. A cluster of tyrosine residues in TRGV5 (Y38, Y42, Y55, Y103, Y113, Y114) and in TRDV1 (Y38, Y42) stabilize the TR γδ – CD1D complex through a multitude of van der Waals/Aromatic interactions both at the Vδ-Vγ interface and at the TR γδ – CD1D interface. Despite their location on different regions, aromatic motifs on TR γδ domains were already described for their key-role in interactions with CD1D (22, 23).

### Choice of dromedary TR γδ cDNA clones for their genomic and functional characteristics

**Figure 2:**
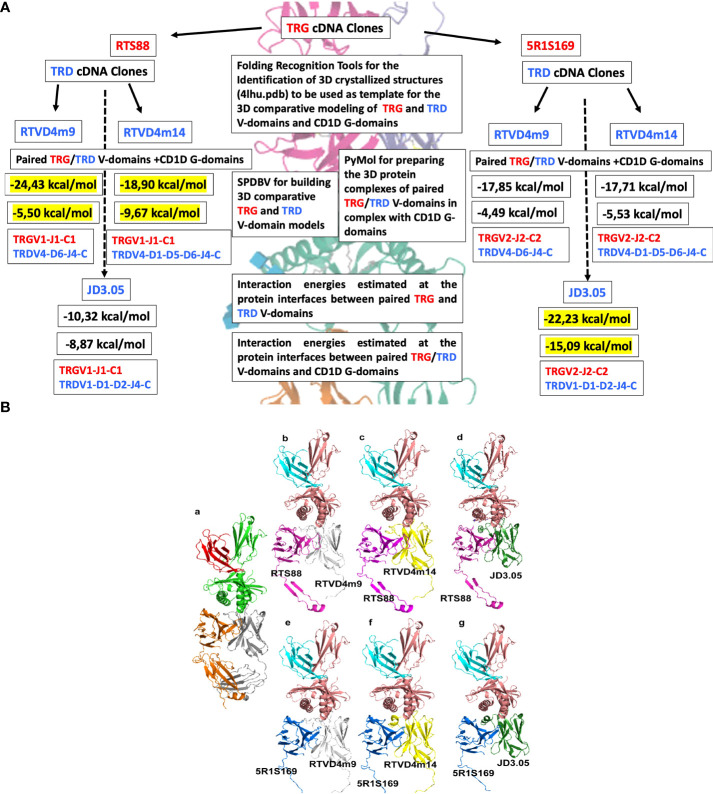
**(A)** Flowchart of the overall *C*. *dromedarius* experimental setting and analyses of TRG and TRD cDNA clones. In correspondence with each combination of the TRG and TRD examined clones (black arrows), the estimated negative energy values (kcal/mol) of the interactions at the protein interfaces between the paired TRG and TRD V-domains, and at the protein interfaces between the paired TRG/TRD V-domains and the CD1D G-domains are shown in succession. Finally, the V-(D)-J-C gene composition of the paired TRG/TRD V-domains corresponding to each combination is indicated. The values in yellow are those deemed most significant because they refer to those closest to the human negative energy value (see **Table S3**). **(B)** *C.dromedarius* TR γδ 3D models in complex with CD1D and B2M. Panel (a) *Homo sapiens* TR γδ 3D model is in orange (TR γ) and gray (TR δ) cartoon, in complex with CD1D (green cartoon) and B2M (red cartoon) as reported in the available crystallized structure 4hlu.pdb. Panel b-g. Different complex poses showing the investigated combination of *C. dromedarius* TRG (RTS88, magenta cartoon; 5R1S169, blue cartoon) and TRD (RTVD4m9, white cartoon; RTVD4m14, yellow cartoon; JD3.05, dark green cartoon) chains in complex with CD1D (salmon cartoon) and of B2M (cyan cartoon).

With the aim of investigating the role of γδ T cells in dromedaries, we took advantage of the simultaneous availability of productively rearranged TRG and TRD cDNA clones from the spleen of one single animal. A flowchart of the overall experimental setting and analyses is reported in **Figure 2A**.

We have chosen five (2 TRG and 3 TRD) cDNA clones to perform a comparison analysis that involves both all the dromedary clones with their human correspondents and all the dromedary clones between themselves. The list of cDNA clones is provided in the **Figure S2**. The choice of TRG cDNA clones was based on the position that the variable TRGV genes occupy in the TRG locus. Two clones RTS88 and 5R1S1S9 contain the variable genes TRGV1 and TRGV2 which are respectively part of the “gene cassettes” TRGC1 and TRGC2 located together with the TRGC5 gene cassette inside the dromedary locus (24). Moreover, clone RTS88 was chosen for this analysis because it harbors four replacements shared by other clones: two in FR1-IMGT, one in CDR2-IMGT, and one in FR3-IMGT. Clone 5R1S169 was chosen since it has three replacements (two in CDR1-IMGT and one in FR2-IMGT), two silent substitutions and its sequence is shared with other clones of the same genealogy (see arrowheads in **Figure S3**) (4).

We also considered genomic characteristics for the choice of TRD clones. RTVD4m9 and RTVD4m14 clones contain the variable TRDV4 gene. This gene, classified according to the initial nomenclature (25), perfectly matches the TRDV3 gene that stands in an inverted transcriptional orientation downstream of the constant TRDC gene within the Sulphur TRA/TRD locus. TRDV4 gene is expressed in *C. dromedarius* equally with TRDV1 in the spleen. The TRDV1 gene is contained in the clone JD3.05 (26).

When considering the alignment of the germline TRDV4 with its mutated cDNA counterpart (clone RTVD4m14), the sequence differs by 10 nucleotide changes (27). Four of these mutations are shared with other clones of the same genealogy **(**see arrowheads in **Figure S4**). That is, the clones grouped in the first genealogy belong to the same recombinative event and share the same CDR3-IMGT. Clone JD3.05 also belongs to a group of hypermutated clones and when compared to its genomic counterpart, it presents small regions of few nucleotides substituted by a gene conversion event (27). **Figure 2A** shows a Flowchart of the comparative analyzes using all the selected TRG and TRD clones.

### 3D modeling of the *C. dromedarius* paired TRG and TRD V-domains in complex with the CD1D G-domains and B2M

*C. dromedarius* TRG and TRD V-domains were modelled by using as a protein template the human 4lhu.pdb TRG chain, which shares with the modelled *C. dromedarius* TRG chain encoded by RTS88 or 5R1S1S9 clones, 47 or 41% of identical amino acids, respectively, or the human 4lhu.pdb TRD chain which shares with the *C. dromedarius* TRD chain encoded by RTVD4m9, RTVD4m14 or JD3.05 clones, 36, 35 or 58% of identical amino acids, respectively and 100% coverage. All *C. dromedarius* TRG and TRD V-domain sequences showed 100% coverage with the corresponding human TRG or TRD V-domains of the crystallized chains. The V-domain pairwise sequence structure alignment between the investigated *C. dromedarius* TRG and TRD and the *Homo sapiens* crystal structure with PDB code 4LHU was obtained by using ClustalW implemented in the Jalview package (28).

The obtained sequence-structure pairwise alignment (**Figure S5A**) was reported in SPDBV alignment panel for guiding/building the 3D comparative model of both TR V-domains, according to validated protocols (8–12). For studying interactions between TRG and TRD V-domains with the G-domains of CD1D in *C. dromedarius* we searched for *C. dromedarius* CD1D and we identified the sequence XP_031291871.1 sharing with the *Homo sapiens* CD1D (4LHU) sequence more than 71% of identical amino acids and 100% coverage. Similarly, we searched for *C. dromedarius* best hit B2M counterpart and the sequence XP_031309022.1 was identified, sharing with the human B2M sequence more than 78% of identical amino acids and 100% coverage (**Figure S5B**).

A 3D comparative model of the *C. dromedarius* CD1D and of B2M was built by using as a protein template the corresponding counterparts taken from 4lhu.pdb starting from the pairwise sequence structure alignment built by CulstalW, as proposed for TRG and TRD chains (**Figures S5A, B** **;** **Figure 2B**). The alpha carbon (Cα) root mean square deviation (RMSD) between the coordinates of the built 3D comparative models and the crystal structure with PDB code 4LHU ranged between 0.54 and 0.57 Å.

### Identification of the CD1D-Restricted γδ T cell receptor in dromedaries

**Figure 3:**
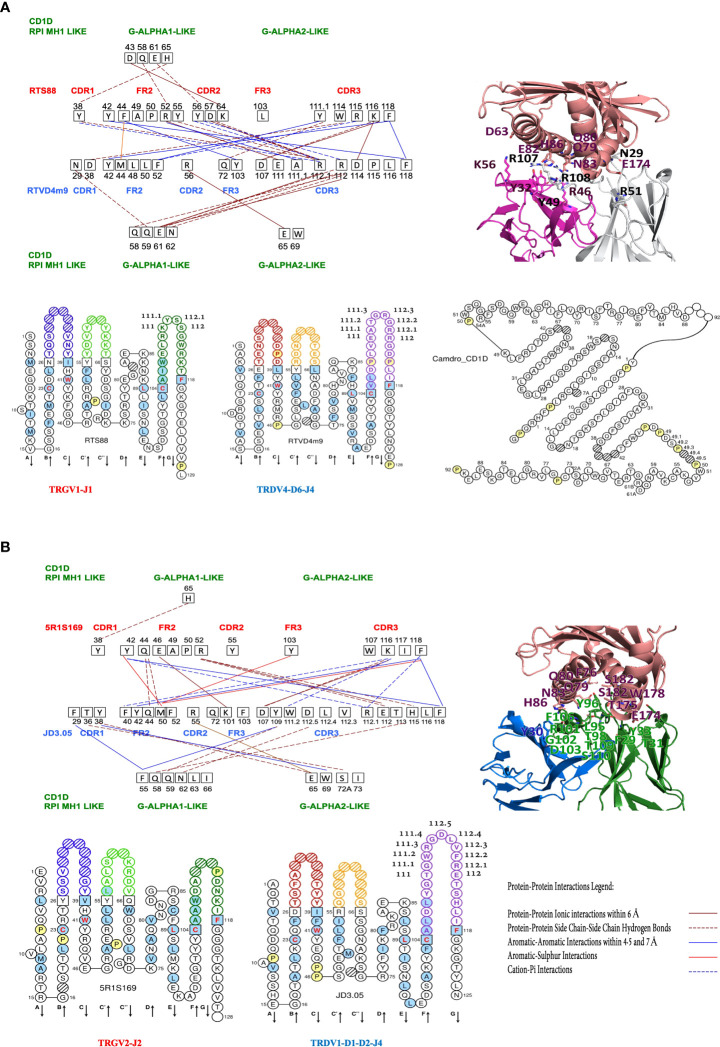
Protein-protein interactions between the Camelus dromedarius FR-IMGT and CDR-IMGT of the TR V-GAMMA (clone RTS88) and V-DELTA (clone RTVD4m9) domains **(A)** and of V-GAMMA (clone 5R1S169) and V-DELTA (clone JD3.05) domains **(B)** and between the V-GAMMA or V-DELTA domains and the Camelus dromedarius G-ALPHA1-LIKE and G-ALPHA2-LIKE domains of the CD1D. On the left, in red the CDR and FR of the TRGV1 **(A)** and TRGV2 **(B)** domains and in blue the CDR and FR of the TRDV4 **(A)** and TRDV1 **(B)** domains; the amino acids and their positions involved in the interactions are shown in black squares. In green the CD1D G-ALPHA1-LIKE and the G-ALPHA2-LIKE domains, with their respective amino acids and their positions involved in the interactions with the V-GAMMA and V-DELTA domains. Amino acids that have no interaction lines, for example RTS88/FR2/49A-50P **(A)** and 5R1S169/FR2/49A-50P **(B)** present hydrophobic interactions within 5 Å (see positions marked in yellow in **Figures S6, S8**). **(A)** Down from left to right, IMGT Collier de Perles of the Camelus dromedarius TRGV1-J1 domain, TRDV4-D6-J4 domain (18, 19) and the G-ALPHA1-LIKE and the G-ALPHA2-LIKE domains of the CD1D, obtained using the IMGT/Collier-de-Perles tool (20). **(B)** Down from left to right, IMGT Collier de Perles of the Camelus dromedarius TRGV2-J2 domain, TRDV1-D1-D2-J4 domain (18, 19) and the Protein-Protein Interactions Legend. **(A, B)** In the upper right, interacting amino acids at the interface of the cited TR gd 3D models with the Homo sapiens CD1D G-ALPHA1-LIKE, and G-ALPHA2-LIKE domains are reported in sticks representation (See also **Figure 2B** and **Table S2**).

The energy calculated for the crystallized 4lhu.pdb (consisting of the *Homo sapiens* TR γδ in complex with CD1D and B2M was used as a reference value. The interaction energies calculated between the TRG and TRD V-domains and between the paired TRG/TRD V-domains and CD1D resulted in a negative value (**Table S3**), confirming that there might be a binding interaction in all the investigated cases. This result is encouraging, also due to the strategy validated by obtaining negative binding energies for the interactions between the *Homo sapiens* TRG and TRD V-domains and CD1D G-domains within the crystal structure with PDB code 4LHU. Notably, the strongest interactions between the investigated *C. dromedarius* TRG and TRD 3D models of the V-domains are observed in the RTS88_RTVD4m9/RTVD4m14-containing protein complexes (-24.43 kcal/mol; -18.90 kcal/mol) (**Figures S6, S7**) and in the 5R1S169_ JD3.05 containing protein complexes (-22.23 kcal/mol), in terms of interaction energies calculated by FoldX Analyze complex assay between TRG and TRD chains (see **Table S3**). The strongest interaction between the paired TRG/TRD V-domains and CD1D G-domains is observed in the RTS88_RTVD4m14-containing protein complexes (-9.67 kcal/mol) (**Figure 2A**; **Figure S7**, **Table S3**) and in the 5R1S69_JD3.05-containing protein complex (-15.09 kcal/mol) (**Figures 2A**, **3B**; **Figure S8**).

In a comparative analysis between humans and dromedaries, the results of the interactions that are established in the TR γδ complex and the CD1D invariant, were quite surprising. In humans, the interactions at the interface between V-DELTA FR2 and V-GAMMA CDR3 are dominant. In turn, V-GAMMA CDR3, while interacting in a milder manner with the three V-DELTA CDR (1–3), recognizes and establishes interaction bonds with G-ALPHA2-LIKE in the region from 61B to 65 (19). The same G-ALPHA2-LIKE, extended to position 69 is recognized by V-DELTA CDR1, CDR2 and FR3 (**Figures 1A, B**; **Table S2** **)**.

In dromedary RTS88_RTVD4m9 containing protein complexes, which correspond to the TRGV1-J1-C1_TRDV4-D6-J4-C pairing (**Figure 3A**), the interactions at the interface between V-GAMMA CDR2 and FR2 and V-DELTA CDR3 are dominant. In turn, it is V-DELTA CDR3 that establishes interaction links with positions Q58, Q59, E61, and N62 of G-ALPHA1-LIKE. In addition, positions D43 and E61, Q58 and H65 of G-ALPHA1-LIKE are recognized by V-GAMMA CDR2, FR2 and CDR1 respectively (**Figure 3A**; **Figure S6**).

In dromedary 5R1S169_JD3.05 containing protein complex, which correspond to the TRGV2-J2-C2_TRDV1-D1-D2-J4-C pairing, the substantial difference compared to the previously described pairing is given by the fact that G-ALPHA2-LIKE is consistently recognized by the V-DELTA CDR1. This result is consistent with the negative energy value (-15.09 kcal/mol), almost similar to the reference value in humans (-15.95 kcal/mol) **Table S3**. Moreover, G-ALPHA2-LIKE is completely ignored by the TRG chain (**Figures 3A, B**; **Figures S6, S8**), with one exception in the position W69 that is involved in the aromatic-aromatic interaction within 4.5 and 7 Å between two tryptophan found in RTS88_RTVD4m14-containing protein complex, which correspond to the TRGV1-J1-C1_TRDV4-D1-D5-D6-J4-C pairing (**Figure 2A**; **Figure S7** **).**

## Discussion

**Figure 4:**
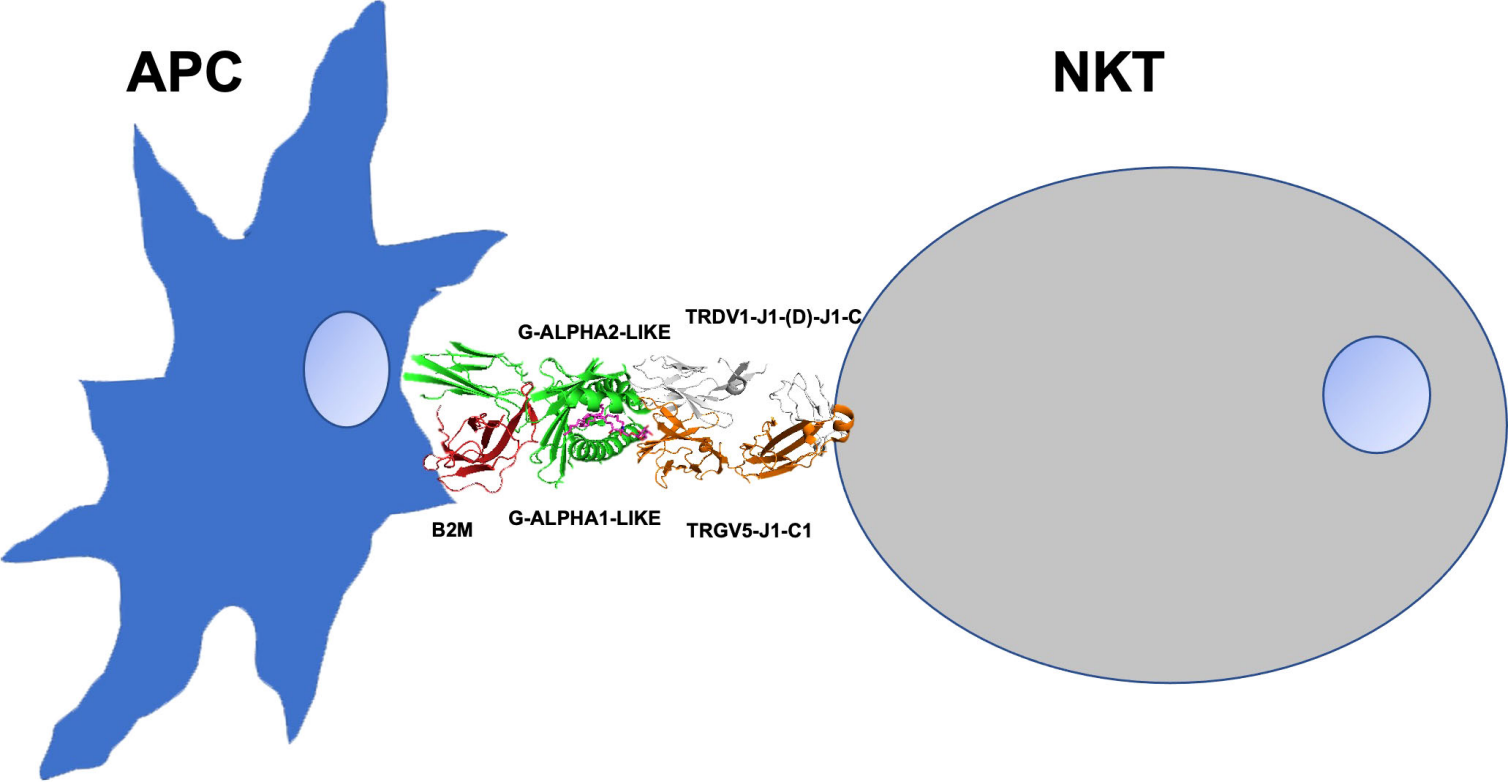
The scheme presents a possible modality of the TR γδ of a human NKT cell that can establish protein interactions with the domains of the CD1D associated with an antigen presenting cell (APC). TR γδ (orange/white), CD1D (green), a-GalCer (magenta) beta-2-microglobulin B2M (red).

In this report, our results inferred from interaction energies values calculated at the protein-protein interface between the crystallized paired *Homo sapiens* TRG and TRD V-domains and between the crystallized *Homo sapiens* TRG/TRD V-domains and CD1D G-domains quantify the strong interactions observed in the crystal structures with PDB code 4LHU and 4LFH presented by Uldrich et al. (1), which postulate the existence in human of γδ T cells that exhibit autoreactivity to CD1D. Natural Killer T (NKT) cells are CD1D-restricted innate-like T cells expressing both T cell receptors and NK cell markers. Invariant NKT cells are the major group of NKT cells in both humans and mice (29). In humans, they express the “invariant” TRAV10-TRAJ18 (Valpha24-JalphaQ) rearrangement, whereas in mice, the “invariant” TR alpha chain of the murine NK1.1^+^ (NKR-P1C, CD161) T cells, which recognize CD1D, results from the TRAV11-TRAJ18 (Valpha14-Jalpha281) rearrangement (IMGT^®^, http://www.imgt.org accessed on 04 March 2022, IMGT Web resources > IMGT Repertoire (IG and TR) > 1. Locus and genes > 7. Gene table: Human: ibid: Mouse). Uldrich et al. (1) highlight that the lineages of αβ T cells and γδ T cells can interact with the same antigen presenting molecule linked to the same ligand. In this report we have quantified the strength of the interaction in the human repertoire of γδ T cells showing the ability to bind to the CD1D. This γδ T cell population expresses TRGV5-TRGJ1 and TRDV1-(D)-TRDJ1 chains (**Figures 1A, B** **)**. Here, in agreement with the proposal for the designation of the natural killer T cell subset as γδ NKT (30), we hypothesize a possible modality with which the TR γδ of a human NKT cell can establish protein interactions with the domains of the CD1D associated with an antigen presenting cell (APC) (see the scheme in **Figure 4** **)**. We also hypothesize that a similar set of interactions might be conserved in the dromedary repertoire of γδ T cells and with this aim we modelled the possible paired TRG/TRD chains in complex with CD1D by using the human TRG/TRD-CD1D complex. Our results showed that interactions between V-DELTA FR2-IMGT and V-GAMMA CDR3-IMGT are predominant in *Homo sapiens* while interactions between V-GAMMA FR2-IMGT and V-DELTA CDR3-IMGT are predominant in *Camelus dromedarius*. This different combination depends both on the genomic characteristics of human FR2 compared to those of camel FR2 but also on the exceptional length of V-DELTA CDR3 in camel (9 or 12 amino acids involved in interactions in camel versus only two in human) (**Figures 1** and **3**). In other TRD clones an even greater length of V-DELTA CDR3 was observed (37 amino acids in clone SC19 of which 10 amino acids are involved in interactions) (manuscript in preparation) (26). The calculation of the interaction energies at the protein-protein interface between the paired *C. dromedarius* TRG and TRD V-domains and between the *C. dromedarius* TRG/TRD V-domains and CD1D G-domains, compared to the interaction energies estimated in the available *Homo sapiens* TRG/TRD-CD1D crystallized structure (1) has allowed to deduce the most efficient pairing of the TRG and TRD chains (-24,43 kcal/mol in the TRGV1-J1-C1 paired to TRDV4-D6-J4-C complex) (underlined in bold in **Table S3**) (**Figure 2A**). Furthermore, we hypothesize that TRGV2-J2-C2 paired to TRDV1-D1-D2-J4-C complex (-15,09 kcal/mol underlined in bold in **Table S3**) (**Figure 2A**) may be the best candidate for producing CD1D mediated reactivity. Notably, the Hydrogen-π interaction between histidine (H65 in G-ALPHA1-LIKE) and aromatic amino acid (Y38 in V-GAMMA CDR1-IMGT) is constantly present in both humans and camels (**Figures 1** and **3**, **Figure S7**) and histidine seems to play a key role in the stability of the TRG/TRD-CD1D complex (23).

In summary, the energetically stabilized protein complexes proposed in our experimental setting and analyses of TRG and TRD cDNA clones, led us to hypothesize the existence of a population of CD1D-restricted γδ T cells in the dromedaries, similarly to what observed by Uldrich et al. in *H. sapiens* (1). Recently, Rice et al. (31) reported about a gamma/delta TR docking to the MR1 antigen presenting molecule, showing that the TR was able to bind to the side of the MR1 antigen binding pocket declaring that the “Characterization of one Vδ3Vγ8 TCR clone showing MR1 reactivity was independent of the presented antigen. Determination of two Vδ3Vγ8 TCR-MR1-antigen complex structures revealed a recognition mechanism by the Vδ3 TCR chain mediated by specific contacts to the side of the MR1 antigen-binding groove, representing a previously uncharacterized MR1 docking topology”. If we compare the pairing human Vδ3Vγ8 TCR-MR1-antigen complex structure published by Rice et al. (31) with the pairing human Vδ1Vγ5 TCR-CD1D published by Uldrich et al. (1), we observed that the investigated TCR bind at different regions of MHC-/CD1D-like molecules and we cannot exclude that a similar MR1-TCRγδ alternate docking mechanism can be observed also in dromedaries.

Notably, in our analyzes, the pairing consists of Vδ1Vγ5 TCR-CD1D-antigen complex and we retain that it is more appropriate due to the peculiarities of Vδ1 in humans, emphasized in our previous works, compared to those found in camelids (25, 26). Notably, the investigated dromedaries δ domains share the highest % of identical residues with the corresponding δ domains observed in the human Vδ1Vγ5 TCR-CD1D-antigen complex crystallized by Uldrich et al. (1)

In conclusion, we predicted similarities of *Camelus dromedarius* gamma/delta TR cDNA sequences with *Homo sapiens* CD1D specific gamma/delta NKT TR and then, using 3D molecular modelling, we mapped the camelid sequences to the previously solved crystal structure of *Homo sapiens* CD1D-gamma/delta NKT TR to determine predicted docking mechanism and amino acid contacts. Our standardized analysis using the IMGT unique numbering for V and G domains for sequences and structures, lays the groundwork for future comparative analysis into the role of dromedary, and other non-human species, gamma/delta TR within respective immune systems.

## Data availability statement

The original contributions presented in the study are included in the article/**Supplementary Material**. Further inquiries can be directed to the corresponding author.

## Ethics statement

The animal study was reviewed and approved by committee indicated in previous studies already published, because the dromedary cDNAs used in this study were taken from the public database.

## Author contributions

Conceptualization, GL, CLP, RA and SC; Methodology, GL, VT, and CLP; Investigation, GL, VT, CLP and SC; Writing—original draft preparation, VT, CLP, and SC; Writing—review and editing, CLP, SM, M-PL and SC; Supervision M-PL and SC; Resources and funding acquisition, RA and SC. All authors contributed to the article and approved the submitted version.

## Funding

The financial support of the University of Bari and the University of Salento is gratefully acknowledged. This research was funded by the “Contributo di Ateneo per progetti competitivi, residuo fondo anno 2015 (University of Bari) and Pon – Ricerca e innovazione 2014-2020 – Progetto AIM 1807508 – attività 1 – linea 1 (Department of Biology, University of Bari).

## Acknowledgments

The authors would like to acknowledge Angela Pala for the technical support and assistance in the manuscript preparation.

## Conflict of interest

The authors declare that the research was conducted in the absence of any commercial or financial relationships that could be construed as a potential conflict of interest.

## Publisher’s note

All claims expressed in this article are solely those of the authors and do not necessarily represent those of their affiliated organizations, or those of the publisher, the editors and the reviewers. Any product that may be evaluated in this article, or claim that may be made by its manufacturer, is not guaranteed or endorsed by the publisher.
