# Supplementary material for: 3D structures inferred from cDNA clones identify the CD1D-Restricted γδ T cell receptor in dromedaries
Source: Front Immunol. 2022 Aug 9;13:928860. doi: 10.3389/fimmu.2022.928860 (PMC9396240; doi:10.3389/fimmu.2022.928860)
Supplement: Supplementary file 1 [file DataSheet_1.pdf]

Supplementary Figures and Tables legends

Supplementary Tables legends

Table S1.
List of the inter-chain and inter-protein interactions within the crystallized human protein TR gamma/delta in complex with the human antigen-presenting glycoprotein CD1D and B2M. The letter "A" indicates the antigen-presenting glycoprotein CD1D. The letters G and D indicate the human TR gamma and delta chains, respectively. The letter B indicates B2M.

Table S2.
Correspondence between the IMGT and PDB numbering of the amino acid positions in the protein-protein interactions between the TRGV5-J1 or TRDV1-J1 chains and the G-ALPHA1-LIKE and G-ALPHA2-LIKE domains of the antigen presenting glycoprotein CD1D in 4hlu.pdb.

Table S3.
Interaction energies estimated at the protein interface between TR gamma and TR delta chains or between the reported TR gamma/delta and CD1D. The most significant negative energy values in *Camelus dromedarius* in comparison with the corresponding (4hlu.pdb) ones in *Homo sapiens* are in bold.

Supplementary Figures legends

Figure S1.
List of the protein-protein interactions of 4lhu.pdb reported in **Supplementary Table 1** as deduced using the IMGT numbering (Lefranc et al., 2005). The positions of the interacting amino acid of TRG (red), of TRD (blue) and of RPI MH1 LIKE (green) chains are shown.

Figure S2.
List of the *C. dromedarius* TRG and TRD clones.

Figure S3.
Mutated TRGV2 cDNA sequences from the adult dromedary spleen. cDNA sequences (Accession No. JF792633–JF792673) are compared with the genomic TRGV2⁄01 and TRGJ2-2⁄01 (Accession No. JN172913) sequences. Only the mutated codons are shown; leader, FR-IMGT and CDR-IMGT are indicated and delimited by small vertical lines. Dashes indicate identity with the reference sequence, whereas mutations are highlighted as nucleotides. Capital letters in bold indicate AA changes. Codon positions that display multiple independent changes are underlined. The two groups of 7 and 8 (square parenthesis) cDNA sets consist of clones sharing the same V-J rearrangement.
This figure is presented with the approval of the authors (Ciccarese et al., 2014).

Figure S4.
Mutant cDNA sequences from adult dromedary lymphoid tissues. Two series of full-length cDNA sequences were obtained from RT-PCR of dromedary spleen and blood RNA. All cDNA sequences were compared with the genomic TRDV4 and TRDJ4 sequences. For the rearranged CDR3-IMGT, only amino acids at positions 105, 116 and 117 were included in the analysis. Only the codons affected by mutations are shown; the tandem mutations are underlined. Leader region, FR and CDR are labeled. Dots indicate identity with reference sequence. For each tissue two groups of related mutant sequences are indicated by a vertical bar, as are the related mutations by different colors. To simplify the reading, the colored changes are excluded from counting. Asterisks indicate that only one of related mutations is included in counting. A tally of the number of mutations and of tandem

mutations is given. Vertical small pipes separate amino acids that are not contiguous in the sequences. In squares are shown codon positions (8, 18, 54 and 78) that display multiple independent changes. This figure is presented with the approval of the authors (Antonacci et al., 2011).

Figure S5.
A. Sequence-structure alignment of the investigated *C. dromedarius* TRG and TRD chains with human TRG and TRD chains from the crystallized structures 4lhu.pdb (Uldrich et al., 2013) and 1hxm.pdb (Xu et al., 2011). **B.** Sequence-structure pairwise alignment of the human CD1D (from 4lhu.pdb) and B2M (from 4lhu.pdb) with their closest homologues in *C. dromedarius*.

Figure S6.
List of the protein-protein interactions in dromedary RTS88_RTVD4m9 containing protein complex as deduced using the IMGT numbering (Lefranc et al., 2005). The positions of the interacting amino acid of TRG (red), of TRD (blue) and of RPI MH1 LIKE (green) chains are shown.

Figure S7.
List of the protein-protein interactions in dromedary RTS88_RTVD4m14 containing protein complex as deduced using the IMGT numbering (Lefranc et al., 2005). The positions of the interacting amino acid of TRG (red), of TRD (blue) and of RPI MH1 LIKE (green) chains are shown.

Figure S8.
List of the protein-protein interactions in dromedary 5R1S169_JD3.05 containing protein complex as deduced using the IMGT numbering (Lefranc et al., 2005). The positions of the interacting amino acid of TRG (red), of TRD (blue) and of RPI MH1 LIKE (green) chains are shown.

References

- Lefranc MP, Pommié C, Kaas Q, Duprat E, Bosc N, Guiraudou D, et al. IMGT unique numbering for immunoglobulin and T cell receptor constant domains and Ig superfamily C-like domains. *Dev Comp Immunol.* (2005) 29:185-203. doi.org/10.1016/j.dci.2004.07.003
- Ciccarese S, Vaccarelli G, Lefranc MP,Tasco G, Consiglio A, Casadio R et al._Characteristics of the somatic hypermutation in the *Camelus dromedarius* T cell receptor gamma (TRG) and delta (TRD) variable domains. *Dev Comp Immunol.* (2014) 46: 300-313. doi.org/10.1016/j.dci.2014.05.001
- Antonacci, R., Mineccia, M., Lefranc, MP., Ashmaoui, H.M.E., Lanave, C., Piccinni, B., et al. Expression and genomic analyses of Camelus dromedarius T cell receptor delta (TRD) genes reveal a variable domain repertoire enlargement due to CDR3 diversification and somatic mutation. *Molecular Immunology* (2011) 48, 1384-1396.
- Uldrich AP, Le Nours J, Pellicci DG, Gherardin NA, McPherson KG, Lim TR, et al. CD1d-lipid antigen recognition by the gδ TCR. *Nat Immunol.* (2013) 14: 1137-1145.doi.org/10.1038/ni.2713
- Xu B, Pizarro JC, Holmes MA, McBeth C, Groh V, Spies T, Strong RK. Crystal structure of a gammadelta T-cell receptor specific for the human MHC class I homolog MICA. *Proc Natl Acad Sci U S A* (2011) 108(6), 2414–9.

Supplementary Tables

Supplementary Table S1

Protein-Protein Hydrophobic Interactions

Rasmol Jmol [help]

4hlu.rep.pdb

Hydrophobic Interactions within 5 Angstroms

Position	Residue	Chain	Position	Residue	Chain
13	LEU	A	56	PHE	B
15	ILE	A	54	LEU	B
15	ILE	A	56	PHE	B
15	ILE	A	62	PHE	B
31	TRP	A	63	TYR	B
36	TYR	D	110	TYR	G
38	PHE	D	101	TRP	G
40	TYR	D	115	PHE	G
48	MET	D	115	PHE	G
48	MET	D	97	TYR	G
50	PHE	D	106	PRO	G
50	PHE	D	114	LEU	G
58	PHE	A	32	TRP	D
66	LEU	A	32	TRP	D
69	ILE	A	102	LEU	D
93	PHE	D	47	PRO	G
107	LEU	D	101	TRP	G
107	LEU	D	115	PHE	G
107	LEU	D	39	TYR	G
109	PHE	D	115	PHE	G
109	PHE	D	39	TYR	G
109	PHE	D	46	ALA	G
109	PHE	D	47	PRO	G
117	ALA	A	60	TRP	B
130	PHE	D	148	LEU	G
132	MET	D	139	PHE	G
132	MET	D	155	LEU	G
139	ALA	D	139	PHE	G
139	ALA	D	155	LEU	G
153	TRP	A	102	LEU	D
153	TRP	A	111	TYR	G
160	TRP	A	102	LEU	D
160	TRP	A	32	TRP	D
160	TRP	A	36	TYR	D
160	TRP	A	99	PRO	D
163	PHE	D	157	LEU	G
163	PHE	D	194	MET	G
166	ALA	D	196	PHE	G
166	ALA	D	198	TRP	G
167	PRO	A	33	TRP	D
175	TYR	D	198	TRP	G

178	VAL	D	155	LEU	G
178	VAL	D	196	PHE	G
178	VAL	D	198	TRP	G
180	LEU	D	139	PHE	G
180	LEU	D	155	LEU	G
180	LEU	D	157	LEU	G
180	LEU	D	196	PHE	G
190	TRP	A	14	PRO	B
195	PRO	A	99	MET	B
203	LEU	A	99	MET	B
205	VAL	A	99	MET	B
236	LEU	A	10	TYR	B
236	LEU	A	26	TYR	B
237	PRO	A	26	TYR	B
237	PRO	A	65	LEU	B
239	ALA	A	65	LEU	B
239	ALA	A	67	TYR	B
244	TYR	A	10	TYR	B

NO PROTEIN-PROTEIN DISULPHIDE BRIDGES FOUND

Protein-Protein Main Chain-Main Chain Hydrogen Bonds

Rasmol **Jmol** [help]

[View the original hbond output]

4hlu.rep.pdb

DONOR				ACCEPTOR					PARAMETERS			
**POS**	**CHAIN**	**RES**	**ATOM**	**POS**	**CHAIN**	**RES**	**ATOM**	**MO**	**Dd-a**	**Dh-a**	**A(d-H-N)**	**A(a-O=C)**
0	B	MET	N	119	A	GLN	O	-	2.87	9.99	999.99	999.99
140	G	LEU	N	133	D	LYS	O	-	3.20	2.25	166.79	144.96

Dd-a = Distance Between Donor and Acceptor
Dh-a = Distance Between Hydrogen and Acceptor
A(d-H-N) = Angle Between Donor-H-N
A(a-O=C) = Angle Between Acceptor-O=C
MO = Multiple Occupancy
Note that angles that are undefined are written as 999.99

Protein-Protein Main Chain-Side Chain Hydrogen Bonds

Rasmol **Jmol** [help]

[View the original hbond output]

4hlu.rep.pdb

DONOR				ACCEPTOR					PARAMETERS			
**POS**	**CHAIN**	**RES**	**ATOM**	**POS**	**CHAIN**	**RES**	**ATOM**	**MO**	**Dd-a**	**Dh-a**	**A(d-H-N)**	**A(a-O=C)**
62	A	GLN	NE2	31	D	SER	O	1	3.01	2.12	140.39	117.61
62	A	GLN	NE2	31	D	SER	O	2	3.01	3.25	67.37	117.61
97	A	GLN	NE2	60	B	TRP	O	1	3.05	2.00	178.10	114.33
97	A	GLN	NE2	60	B	TRP	O	2	3.05	3.65	48.36	114.33
120	A	GLY	N	31	B	HIS	NE2	-	3.17	3.31	73.57	999.99
153	A	TRP	NE1	101	D	GLY	O	-	3.48	2.94	119.95	158.33
160	A	TRP	NE1	34	D	SER	O	-	2.98	2.13	155.90	111.80
168	A	GLN	NE2	31	D	SER	O	1	3.00	2.25	126.03	143.69
168	A	GLN	NE2	31	D	SER	O	2	3.00	3.26	66.63	143.69
193	A	ARG	N	98	B	ASP	OD1	-	3.27	2.41	147.07	86.63
193	A	ARG	N	98	B	ASP	OD2	-	3.31	2.62	126.88	85.16
209	A	SER	OG	12	B	ARG	O	-	2.95	9.99	999.99	135.96
246	A	ARG	NH1	99	B	MET	O	1	2.79	1.98	130.58	155.25
246	A	ARG	NH1	99	B	MET	O	2	2.79	3.11	62.27	155.25
10	B	TYR	OH	237	A	PRO	O	-	2.83	9.99	999.99	151.70
33	B	SER	N	95	A	GLU	OE1	-	2.90	1.92	166.44	134.37
98	B	ASP	OD2	193	A	ARG	O	1	3.16	3.63	56.05	105.53
98	B	ASP	OD2	193	A	ARG	O	2	3.16	3.20	78.01	105.53
99	B	MET	N	192	A	SER	OG	-	2.81	1.84	158.27	999.99
33	D	TRP	N	168	A	GLN	OE1	-	2.94	2.04	150.99	125.34
40	D	TYR	OH	113	G	LYS	O	-	2.84	9.99	999.99	124.12
107	D	LEU	N	39	G	TYR	OH	-	3.26	2.79	109.80	999.99
111	D	LYS	NZ	44	G	GLY	O	-	2.80	9.99	999.99	160.90
134	D	ASN	ND2	138	G	ILE	O	1	2.61	3.27	43.84	155.49
134	D	ASN	ND2	138	G	ILE	O	2	2.61	1.63	156.21	155.49
11	G	THR	OG1	45	D	SER	O	-	3.12	9.99	999.99	156.42
37	G	HIS	NE2	103	D	ASN	O	-	2.91	2.13	147.42	154.20
49	G	ARG	NH1	104	D	THR	O	1	2.88	3.26	60.01	145.80
49	G	ARG	NH1	104	D	THR	O	2	2.88	1.93	154.27	145.80
97	G	TYR	OH	46	D	LYS	O	-	2.74	9.99	999.99	137.44
101	G	TRP	NE1	102	D	LEU	O	-	3.29	3.25	84.31	145.17
101	G	TRP	NE1	105	D	ASP	O	-	3.06	2.23	151.14	126.92
113	G	LYS	NZ	105	D	ASP	O	-	2.65	9.99	999.99	153.90

Dd-a = Distance Between Donor and Acceptor
Dh-a = Distance Between Hydrogen and Acceptor
A(d-H-N) = Angle Between Donor-H-N
A(a-O=C) = Angle Between Acceptor-O=C
MO = Multiple Occupancy
Note that angles that are undefined are written as 999.99

Protein-Protein Side Chain-Side Chain Hydrogen Bonds

Rasmol **Jmol** [help]

[View the original hbond output]

4hlu.rep.pdb

DONOR				ACCEPTOR					PARAMETERS			
POS	CHAIN	RES	ATOM	POS	CHAIN	RES	ATOM	MO	Dd-a	Dh-a	A(d-H-N)	A(a-O=C)
17	A	SER	OG	33	B	SER	OG	-	3.17	9.99	999.99	999.99
36	A	GLN	NE2	53	B	ASP	OD2	1	2.68	1.68	157.07	999.99
36	A	GLN	NE2	53	B	ASP	OD2	2	2.68	3.06	59.24	999.99
39	A	SER	OG	53	B	ASP	OD2	-	2.83	9.99	999.99	999.99
68	A	HIS	ND1	35	G	TYR	OH	-	3.25	2.66	123.87	999.99
97	A	GLN	OE1	31	B	HIS	NE2	1	2.86	2.18	119.48	999.99
97	A	GLN	OE1	31	B	HIS	NE2	2	2.86	2.84	80.34	999.99
152	A	LYS	NZ	110	G	TYR	OH	-	2.92	9.99	999.99	999.99
192	A	SER	OG	98	B	ASP	OD1	-	2.84	9.99	999.99	999.99
193	A	ARG	NH1	98	B	ASP	OD2	1	3.24	3.13	85.94	999.99
193	A	ARG	NH1	98	B	ASP	OD2	2	3.24	3.33	75.98	999.99
238	A	ASN	OD1	24	B	ASN	ND2	1	2.91	3.07	71.08	999.99
238	A	ASN	OD1	24	B	ASN	ND2	2	2.91	1.98	143.45	999.99
12	B	ARG	NH1	240	A	ASP	OD2	1	2.10	1.22	133.73	999.99
12	B	ARG	NH1	240	A	ASP	OD2	2	2.10	2.42	59.33	999.99
24	B	ASN	ND2	238	A	ASN	OD1	1	2.91	2.15	126.81	999.99
24	B	ASN	ND2	238	A	ASN	OD1	2	2.91	3.25	62.06	999.99
31	B	HIS	NE2	97	A	GLN	OE1	-	2.86	2.00	163.47	999.99
33	B	SER	OG	17	A	SER	OG	-	3.17	9.99	999.99	999.99
33	B	SER	OG	95	A	GLU	OE1	-	3.12	9.99	999.99	999.99
33	B	SER	OG	95	A	GLU	OE2	-	2.76	9.99	999.99	999.99
53	B	ASP	OD2	36	A	GLN	NE2	1	2.68	2.96	64.64	999.99
53	B	ASP	OD2	36	A	GLN	NE2	2	2.68	1.72	146.50	999.99
60	B	TRP	NE1	122	A	ASP	OD1	-	2.93	2.04	166.51	999.99
60	B	TRP	NE1	122	A	ASP	OD2	-	3.01	2.36	128.28	999.99
36	D	TYR	OH	156	A	GLU	OE2	-	2.89	9.99	999.99	999.99
42	D	GLN	NE2	41	G	HIS	ND1	1	3.12	2.19	146.74	999.99
42	D	GLN	NE2	41	G	HIS	ND1	2	3.12	3.50	60.79	999.99
42	D	GLN	NE2	97	G	TYR	OH	1	3.34	3.55	69.91	999.99
42	D	GLN	NE2	97	G	TYR	OH	2	3.34	2.42	145.09	999.99
106	D	LYS	NZ	63	G	GLU	OE1	-	2.85	9.99	999.99	999.99
143	D	LYS	NZ	145	G	GLU	OE2	-	2.75	9.99	999.99	999.99
143	D	LYS	NZ	149	G	GLN	OE1	-	2.84	9.99	999.99	999.99
35	G	TYR	OH	68	A	HIS	ND1	-	3.25	9.99	999.99	999.99
41	G	HIS	ND1	42	D	GLN	NE2	-	3.12	2.63	116.16	999.99
52	G	TYR	OH	64	A	GLU	OE2	-	2.97	9.99	999.99	999.99
97	G	TYR	OH	42	D	GLN	NE2	-	3.34	9.99	999.99	999.99
110	G	TYR	OH	156	A	GLU	OE1	-	2.91	9.99	999.99	999.99
117	G	SER	OG	47	D	GLU	OE2	-	3.37	9.99	999.99	999.99

Dd-a = Distance Between Donor and Acceptor
Dh-a = Distance Between Hydrogen and Acceptor
A(d-H-N) = Angle Between Donor-H-N
A(a-O=C) = Angle Between Acceptor-O=C
MO = Multiple Occupancy
Note that angles that are undefined are written as 999.99

Protein-Protein Ionic Interactions

[help]
Rasmol Jmol

4hlu.rep.pdb

Ionic Interactions within 6 Angstroms

Position	Residue	Chain	Position	Residue	Chain
48	ARG	A	53	ASP	B
95	GLU	A	31	HIS	B
106	LYS	D	63	GLU	G
143	LYS	D	145	GLU	G
152	LYS	A	58	GLU	D
156	GLU	A	53	ARG	D
182	LYS	D	159	GLU	G
193	ARG	A	98	ASP	B
207	HIS	A	98	ASP	B
240	ASP	A	12	ARG	B

Protein-Protein Aromatic-Aromatic Interactions

Rasmol Jmol [help]

4hlu.rep.pdb

Aromatic-Aromatic Interactions within 4.5 and 7 Angstroms

Residue	Position	Chain	Residue	Position	Chain	D(centroid-centroid)	Dihedral Angle
31	TRP	A	63	TYR	B	6.11	6.85
36	TYR	D	110	TYR	G	6.51	117.01
38	PHE	D	101	TRP	G	5.25	70.86
40	TYR	D	115	PHE	G	5.47	86.32
58	PHE	A	32	TRP	D	5.33	133.92
109	PHE	D	115	PHE	G	6.20	80.46
109	PHE	D	39	TYR	G	6.00	101.00
153	TRP	A	111	TYR	G	6.09	92.94
160	TRP	A	32	TRP	D	5.22	59.82
175	TYR	D	198	TRP	G	6.76	82.54
244	TYR	A	10	TYR	B	4.53	157.32

Protein-Protein Aromatic-Sulphur Interactions

Rasmol Jmol [help]

4hlu.rep.pdb

Aromatic-Sulphur Interactions within 5.3 Angstroms

Position	Residue	Chain	Position	Residue	Chain	D(Centroid-Sulphur)	Angle
97	TYR	G	48	MET	D	3.86	170.92
115	PHE	G	48	MET	D	3.87	152.37
163	PHE	D	194	MET	G	4.59	64.86

Protein-Protein Cation-Pi Interactions

Rasmol Jmol [help]

4hlu.rep.pdb

Cation-Pi Interactions within 6 Angstroms

Position	Residue	Chain	Position	Residue	Chain	D(cation-Pi)	Angle
50	PHE	D	112	LYS	G	5.76	121.24
110	TYR	G	152	LYS	A	4.85	71.32
110	TYR	G	53	ARG	D	3.79	170.16

Supplementary Table S2

G-ALPHA 1-LIKE		TRGV5-J1		TRDV1-J1	
**IMGT**	**PDB**	**IMGT**	**PDB**	**IMGT**	**PDB**
F (PHE) 55	F (PHE) 58	----	----	W (TRP) 29	W (TRP) 32
E (GLU) 61	E (GLU) 64	Y (TYR) 55	Y (TYR) 52	----	----
H (HIS) 65	H (HIS) 68	Y (TYR) 38	Y (TYR) 35	----	----

G-ALPHA 2-LIKE		TRGV5-J1		TRDV1-J1	
**IMGT**	**PDB**	**IMGT**	**PDB**	**IMGT**	**PDB**
K (LYS) 61B	K (LYS) 152	Y (TYR) 113	Y (TYR) 110	E (GLU) 67	E (GLU) 58
W (TRP) 62	W (TRP) 153	Y (TYR) 114	Y (TYR) 111	----	----
E (GLU) 65	E (GLU) 156	Y (TYR) 113	Y (TYR) 110	R (ARG) 55	R (ARG) 53
E (GLU) 65	E (GLU) 156	----	----	Y (TYR) 38	Y (TYR) 36
W (TRP) 69	W (TRP) 160	----	----	W (TRP) 29	W (TRP) 32

Supplementary Table S3. Interaction energies estimated at the protein interface between TR γ and TR δ chains or between the reported TR γδ and CD1D.

Pdb	4lhu.pdb		RTS88_RTVD4m9		RTS88_RTVD4m14		RTS88_JD3.05		5R1S169_RTVD4m9		5R1S169_RTVD4m14		5R1S169_JD3.05	
Group1	G	GD	G	GD	G	GD	G	GD	G	GD	G	GD	G	GD
Group2	D	C (A)	D	C	D	C	D	C	D	C	D	C	D	C
Interaction Energy	-42,08	-15,95	**-24,43**	**-5,50**	**-18,90**	**-9,67**	-10,32	-8,87	-17,85	-4,49	-17,71	-5,53	**-22,23**	**-15,09**
IntraclashesGroup1	10,82	9,75	7,34	22,27	8,17	9,01	3,56	17,29	3,79	18,57	13,98	7,35	4,29	8,94
IntraclashesGroup2	6,99	18,48	13,06	8,29	8,42	17,80	9,03	5,96	13,12	7,49	8,51	24,11	3,20	9,04
Backbone Hbond	-6,81	-1,94	-4,84	-1,05	-3,51	-1,09	-2,42	-0,31	-5,28	-1,13	-5,79	-0,59	-2,14	-0,53
Sidechain Hbond	-10,46	-8,06	-10,52	-9,09	-6,31	-7,82	-4,64	-4,56	-5,31	-10,57	-6,47	-5,01	-10,03	-6,34
Van der Waals	-35,78	-14,05	-22,42	-10,75	-27,64	-10,98	-24,19	-9,56	-22,01	-10,20	-24,21	-8,92	-22,48	-12,04
Electrostatics	-3,08	-0,57	-2,19	-2,40	-2,35	-1,94	-2,63	-0,62	-2,04	-2,13	-3,48	-0,52	-2,93	-0,04
Solvation Polar	39,59	15,37	27,66	17,77	34,68	15,51	32,28	11,98	27,86	17,81	33,26	12,42	28,10	12,53
Solvation Hydrophobic	-50,04	-18,74	-29,12	-10,54	-35,16	-12,71	-31,48	-12,44	-28,79	-9,27	-30,13	-10,22	-29,36	-15,94
Van der Waals clashes	0,53	0,63	0,37	0,51	0,83	0,41	4,34	0,38	0,27	0,33	1,18	0,76	1,33	0,21
entropy sidechain	14,77	9,40	9,01	8,87	11,65	7,00	11,28	4,55	9,08	10,32	10,81	5,31	9,97	6,85
entropy mainchain	10,31	1,96	6,53	1,03	8,55	1,78	7,17	1,71	7,31	0,51	6,72	0,71	6,15	0,29
torsional clash	0,13	0,04	1,50	0,28	0,38	0,12	0,35	0,03	1,39	0,24	0,44	0,10	0,22	0,01

backbone clash	2,15	2,37	2,49	1,55	2,55	1,16	2,88	1,26	1,65	1,64	2,53	1,57	2,10	2,46
helix dipole	-0,25	0,14	-0,02	0,15	-0,02	0,25	0,00	0,05	0,00	0,04	0,00	0,03	-0,34	-0,19
electrostatic kon	-1,09	-0,15	-0,47	-0,63	-0,05	-0,26	-0,40	-0,10	-0,41	-0,54	-0,43	0,24	-0,74	0,04
Entropy Complex	2,38	2,38	2,38	2,38	2,38	2,38	2,38	2,38	2,38	2,38	2,38	2,38	2,38	2,38
Number of Residues	801	801	654	654	654	654	642	642	629	629	629	629	617	617
