# Supplementary material for: 3D structures inferred from cDNA clones identify the CD1D-Restricted γδ T cell receptor in dromedaries
Source: Front Immunol. 2022 Aug 9;13:928860. doi: 10.3389/fimmu.2022.928860 (PMC9396240; doi:10.3389/fimmu.2022.928860)
Supplement: Supplementary file 2 [file Image_1.pdf]

4HLU.rep.pdb

1) Protein-protein Ionic Interactions

1.1 Ionic interactions within 6 Angstroms

Chain	Position	Residue	FR/CDR	Chain	Position	Residue	FR/CDR
TRG	68	GLU (E)	FR3	TRD	115	LYS (K)	CDR3

Chain	Position	Residue	FR/CDR	Chain	Position	Residue	G-ALPHA-LIKE
TRD	67	GLU (E)	FR3	RPI MH1 LIKE	61B	LYS (K)	G-ALPHA2
TRD	55	ARG(R)	CDR2	RPI MH1 LIKE	65	GLU (E)	G-ALPHA2

2) Protein-protein Side Chain-Side Chain Hydrogen Bonds

Chain	Position	Residue	FR/CDR	Chain	Position	Residue	FR/CDR
TRG	44	HIS (H)	CDR2	TRD	44	GLN (Q)	FR2
TRG	68	GLU (E)	FR3	TRD	115	LYS (K)	CDR3
TRG	103	TYR (Y)	FR3	TRD	44	GLN (Q)	FR2
TRG	120	SER (S)	FR4	TRD	49	GLU (E)	FR2

Chain	Position	Residue	FR/CDR	Chain	Position	Residue	G-ALPHA-LIKE
TRG	38	TYR (Y)	CDR1	RPI MH1 LIKE	65	HIS (H)	G-ALPHA1
TRG	55	TYR (Y)	CDR2	RPI MH1 LIKE	61	GLU (E)	G-ALPHA1
TRG	113	TYR (Y)	CDR3	RPI MH1 LIKE	61B	LYS (K)	G-ALPHA2
TRG	113	TYR (Y)	CDR3	RPI MH1 LIKE	65	GLU (E)	G-ALPHA2
TRD	38	TYR (Y)	CDR1	RPI MH1 LIKE	65	GLU (E)	G-ALPHA2

3) Protein-Protein Aromatic-Aromatic Interactions

3.1 Aromatic-Aromatic Interactions within 4.5 and 7 Angstrom

Chain	Position	Residue	FR/CDR	Chain	Position	Residue	FR/CDR
TRG	42	TYR (Y)	FR2	TRD	118	PHE (F)	CDR3
TRG	107	TRP(W)	CDR3	TRD	40	PHE (F)	FR2
TRG	113	TYR (Y)	CDR3	TRD	38	TYR (Y)	CDR1
TRG	118	PHE (F)	CDR3	TRD	42	TYR (Y)	FR2
TRG	118	PHE (F)	CDR3	TRD	118	PHE (F)	CDR3

Chain	Position	Residue	FR/CDR	Chain	Position	Residue	G-ALPHALIKE
TRG	114	TYR (Y)	CDR3	RPI MH1 LIKE	62	TRP(W)	G-ALPHA2
TRD	29	TRP(W)	CDR1	RPI MH1 LIKE	55	PHE (F)	G-ALPHA1
TRD	29	TRP(W)	CDR1	RPI MH1 LIKE	69	TRP(W)	G-ALPHA2

4) Protein-Protein Aromatic-Sulphur Interactions

Chain	Position	Residue	FR/CDR	Chain	Position	Residue	FR/CDR
TRG	103	TYR (Y)	FR3	TRD	50	MET (M)	FR2
TRG	118	PHE (F)	CDR3	TRD	50	MET (M)	FR2

5) Protein-Protein Cation-Pi Interactions

Chain	Position	Residue	FR/CDR	Chain	Position	Residue	FR/CDR
TRG	113	TYR (Y)	CDR3	TRD	55	ARG (R)	CDR2
TRG	115	LYS (K)	CDR3	TRD	52	PHE (F)	FR2

Chain	Position	Residue	FR/CDR	Chain	Position	Residue	G-ALPHA-LIKE
TRG	113	TYR (Y)	CDR3	RPI MH1 LIKE	61B	LYS (K)	G-ALPHA2

6) Protein-Protein Hydrophobic Interactions
6.1 Hydrophobic Interactions within 5 Angstroms

Chain	Position	Residue	FR/CDR	Chain	Position	Residue	FR/CDR
TRG	42	TYR (Y)	FR2	TRD	116	LEU (L)	CDR3
TRG	42	TYR (Y)	FR2	TRD	118	PHE (F)	CDR3
TRG	49	ALA (A)	FR2	TRD	118	PHE (F)	CDR3
TRG	50	PRO (P)	FR2	TRD	118	PHE (F)	CDR3
TRG	50	PRO (P)	FR2	TRD	103	PHE (F)	CDR3
TRG	103	TYR (Y)	FR3	TRD	50	MET (M)	FR2
TRG	107	TRP W)	CDR3	TRD	40	PHE (F)	FR2
TRG	107	TRP W)	FR2	TRD	116	LEU (L)	CDR3
TRG	111A	PRO (P)	CDR3	TRD	52	PHE (F)	FR2
TRG	113	TYR (Y)	CDR3	TRD	38	TYR (Y)	CDR1
TRG	117	LEU (L)	CDR3	TRD	52	PHE (F)	FR2
TRG	118	PHE (F)	CDR3	TRD	42	TYR (Y)	FR2
TRG	118	PHE (F)	CDR3	TRD	50	MET (M)	FR2
TRG	118	PHE (F)	CDR3	TRD	116	LEU (L)	CDR3
TRG	118	PHE (F)	CDR3	TRD	118	PHE (F)	CDR3

Chain	Positio	Residue	FR/CDR	Chain	Positio	Residue	G-ALPHA-LIKE
TRG	114	TYR(Y)	CDR3	RPI MH1 LIKE	62	TRP(W)	G-ALPHA2
TRD	29	TRP(W)	CDR1	RPI MH1 LIKE	69	TRP(W)	G-ALPHA2
TRD	29	TRP(W)	CDR1	RPI MH1 LIKE	54	PHE(F)	G-ALPHA1
TRD	29	TRP(W)	CDR1	RPI MH1 LIKE	63	LEU(L)	G-ALPHA1
TRD	30	TRP(W)	CDR1	RPI MH1 LIKE	75	PRO(P)	G-ALPHA2
TRD	38	TYR (Y)	CDR1	RPI MH1 LIKE	69	TRP(W)	G-ALPHA2
TRD	109	PRO (P)	CDR3	RPI MH1 LIKE	69	TRP(W)	G-ALPHA2
TRD	112A	LEU (L)	CDR3	RPI MH1 LIKE	69	TRP(W)	G-ALPHA2
TRD	112A	LEU (L)	CDR3	RPI MH1 LIKE	66	ILE(I)	G-ALPHA1
