# Supplementary material for: 3D structures inferred from cDNA clones identify the CD1D-Restricted γδ T cell receptor in dromedaries
Source: Front Immunol. 2022 Aug 9;13:928860. doi: 10.3389/fimmu.2022.928860 (PMC9396240; doi:10.3389/fimmu.2022.928860)
Supplement: Supplementary file 3 [file Image_2.pdf]

***Camelus dromedarius* TRG clones**

**>RTS88**
**SSNMEGDKTSITMKVGSSFEMTCDLNTQSVNYIHWYKFGDRTAPRRLFYYDVYYKKTSYDSGIDRKKSRTYE GAEKKNKLLISNLENSDSGTYLCAIWELRKYSSGWRKT FGKGTELIVVPL ASDLDADVSPKPTIFLPSIAEINLHRVGTYLCLLE**

Accession number: JF755952.1
Gene's rearrangement: TRGV1*01-J1-1*01-TRGC1

**>5R1S169**
**EVRLVQPALVMARTRGSTTLPCRTYSSVGYVHWYRQVEGRAPERLLYLALSKRDVQWDSVLRGDKVNAQVNN DGRSCFLSLMKLEKADEGTYYCAAWDAPDNKI FGGGTKLVVT DRGFDADMGPKPTMFLP**

Accession number: JF792640.1
Gene's rearrangement: TRGV2*01-J2-2*01-TRGC2

***Camelus dromedarius* TRD clones**

**>RTVD4m9**
**SAKVTQTSRDQTVASGSEVTLSCTFETENSDPDLYWYRMRPGLSLEFVLYRDNTESLNADFAQGTFSVQHNV AQKTFHLVMSSVRAEDSATYYCVL DPLVEATGRGRRG DPLIFGKGTYLNVEP RKQSVATPS**

Accession number: FN252371.1
Gene's rearrangement: TRDV4-TRDD6-TRDJ4-TRDC

**>RTVD4m14**
**SAKVTLTSRDQTVASGSEVTLPCIFETEYSDPDLYWYRMRPGLFFEFVLYRDNTESLNAVFAQGRFFVQPRL AQKTFHLVMSSVRAEDSATYYCVL EPRYGVISIRGRQ DPLIFGKGTYLNVAP RKQSVATPS**

Accession number: FN252376.1
Gene's rearrangement: TRDV4-TRDD1-D5-D6-TRDJ4-TRDC

**>JD3.05**
**AQTVTQDQPAVSSHEGQTATLKCQFDTSFATYYIFWYEQPPSGEMTFLIRQGSSSGNAKSGRYFIKFRKAEK SLSLTISNLQLEDSAKYFC ALDLYGTGWRGDLVFRETS HLIFGKGTYLN**

Accession number: FN252345
Gene's rearrangement: TRDV1-TRDD1-D2-TRDJ4

**Colors Legend:**

**TRV Variable**
**TRDD Diversity**
**TRJ Joining**
**TRC Constant**
