# Supplementary material for: 3D structures inferred from cDNA clones identify the CD1D-Restricted γδ T cell receptor in dromedaries
Source: Front Immunol. 2022 Aug 9;13:928860. doi: 10.3389/fimmu.2022.928860 (PMC9396240; doi:10.3389/fimmu.2022.928860)
Supplement: Supplementary file 5 [file Image_4.pdf]

		Leader				FR1													CDR1		FR2					CDR2	FR3
		-12	-3	-2	-1	6	7	8	10	12	13	16	17	18	20	22	23	24	27	29	48	49	50	54	55	58	66
		G	G	V	L	Q	T	S	D	T	V	G	S	E	T	P	C	T	T	Y	L	S	L	L	Y	N	S
VD4.14		GGC	GGT	GTG	CTG	CAG	ACG	TCC	GAC	ACA	GTG	GGC	AGT	GAG	ACA	CCC	TGC	ACT	ACC	TAC	CTT	TCT	CTT	CTG	TAC	AAC	TCC
> RTVD4m14	I	..A	...	...	..C	.T.	...	...	...	...	...	..T	...	...	...	...	..T	.T.	...	...	...	.T.	T..	...	...	...	...
RTVD4m17	I	...	...	...	...	...	...	...	...	...	...	..T	...	...	...	...	..T	.T.	...	...	...	...	...	...	...	...	...
RTVD4m18	I	...	...	...	...	...	...	...	...	...	...	..T	...	...	...	...	..T	.T.	G..	...	...	...	...	...	...	...	...
RTVD4m29	I	...	...	...	...	...	...	...	...	...	...	..T	...	...	...	...	..T	.T.	...	...	...	...	...	...	...	...	...
RTVD4m30		...	...	...	...	...	.A.	...	...	...	...	...	...	...	...	...	...	...	...	...	..C	...	...	...	...	.GT	...
RTVD4m16		...	...	...	...	...	...	.T.	...	...	...	...	...	..A	...	...	...	...	...	...	...	...	...	G..	...	...	...
RTVD4m5		...	...	..A	...	...	...	...	...	.G.	...	...	...	...	..T	...	...	...	...	...	...	...	...	..A	...	...	...
RTVD4m26		...	...	...	..T	...	...	..G	T..	...	...	...	...	...	...	...	...	...	...	...	...	...	...	...	...	...	...
RTVD4m10		...	...	...	...	...	...	...	...	...	...	...	...	...	...	...	...	...	...	...	...	...	...	...	...	...	...
RTVD4m25	II	...	...	...	...	...	...	...	...	...	...	...	..A	...	...	...	..T	...	...	A..	..A	...	...	...	..T	...	...
RTVD4m4	II	...	...	...	...	...	...	...	...	...	...	...	...	...	...	...	..T	...	...	A..	...	...	...	...	..T	...	...
SC43	II	...	...	...	...	...	...	...	...	...	...	...	...	...	...	...	..T	...	...	A..	...	...	...	...	..T	...	...
> RTVD4m9	II	...	...	...	...	...	...	...	...	...	...	...	...	...	...	T..	..T	...	...	A..	...	...	...	...	..T	...	...
RTVD4bl7	I	...	...	...	...	...	...	...	..G	...	...	...	...	...	...	...	...	...	...	...	...	...	...	G..	...	...	...
RTVD4bl6	I	...	..C	...	...	...	...	.T.	...	...	.C.	...	...	..A	...	...	...	...	...	...	...	...	...	G..	...	...	...
RTVD4bl21	I	...	...	...	...	...	...	.T.	...	...	...	...	...	..A	...	...	...	...	...	...	...	...	...	G..	...	...	...
RTVD4bl2	I	...	...	...	...	...	...	.T.	...	...	...	...	...	..A	...	...	...	...	...	...	...	...	...	G..	...	...	...
RTVD4bl32	II	...	...	...	...	...	...	...	..G	...	...	...	...	...	...	...	...	...	...	...	...	.T.	...	...	...	...	.G.
RTVD4bl10	II	...	...	...	...	...	...	...	..G	...	...	...	...	...	...	...	...	...	...	...	...	...	.C.	...	...	...	.G.
RTVD4bl15	II	...	...	...	...	...	...	.T.	...	...	...	...	...	..A	...	...	...	...	...	...	...	...	...	...	...	...	.G.
RTVD4bl31	II	...	...	...	...	...	...	.T.	...	...	...	...	...	..A	...	...	...	...	...	...	...	...	...	G..	...	...	.G.

	FR3																						CDR3			FR4
	70	74	75	76	77	78	79	80	81	82	83	84	85	86	90	91	93	95	96	99	100	104	105	116	117	118
	D	G	R	F	S	V	Q	H	N	V	A	Q	K	T	V	M	S	R	A	S	A	C	V	L	I	F
VD4.14	GAT	GGT	AGA	TTT	TCT	GTG	CAG	CAC	AAT	GTG	GCT	CAG	AAA	ACT	GTG	ATG	TCA	AGG	GCT	AGT	GCC	TGT	GTT	CTG	ATA	TTC
RTVD4m14	.T.	...	...	...	.T.	...	...	.C.	.GA	C..	...	...	...	...	...	...	...	...	...	...	...	...	...	...	...	...
RTVD4m17	CT.	A..	...	...	...	..T	A..	...	.G.	...	...	...	...	...	...	..A	...	...	...	.A.	...	...	...	...	...	...
RTVD4m18	.T.	A..	...	...	...	..T	A..	...	.G.	...	...	...	...	...	...	...	...	...	...	...	...	...	...	...	...	...
RTVD4m29	.T.	A..	...	...	...	..T	A..	...	.G.	...	...	...	...	..C	...	...	...	...	...	...	...	...	...	...	...	...
RTVD4m30	...	...	...	...	...	...	...	...	...	...	...	...	...	...	...	...	...	...	A..	...	...	...	...	...	...	...
RTVD4m16	...	...	...	...	...	...	...	...	...	...	..G	...	.G.	...	...	...	...	...	...	...	...	...	...	...	...	...
RTVD4m5	...	...	...	...	...	...	...	...	...	...	...	..C	...	...	.C.	...	...	...	...	...	.G.	...	...	...	..C	...
RTVD4m26	...	...	...	...	...	...	...	...	...	...	...	...	...	...	...	...	...	.C.	...	...	...	...	...	...	...	...
RTVD4m10	...	...	...	...	...	...	...	...	...	...	...	...	...	...	...	...	C..	...	...	...	...	...	..G	...	...	...
RTVD4m25	...	...	.C.	..C	...	...	...	...	...	...	...	...	...	...	...	...	...	...	...	..A	...	...	...	T..	..T	...
RTVD4m4	...	...	.C.	..C	...	...	...	...	...	...	...	...	...	...	...	...	...	...	...	...	...	...	...	T..	..T	...
SC43	...	...	.C.	..C	...	...	...	...	...	...	...	...	...	...	...	...	...	...	...	...	...	...	...	T..	..T	...
RTVD4m9	...	...	.C.	..C	...	...	...	...	...	...	...	...	...	...	...	...	...	...	...	...	...	...	...	T..	..T	...
RTVD4bl7	...	...	...	...	...	.C.	...	...	...	...	..G	.G.	.G.	...	...	...	...	...	...	...	...	...	...	...	...	...
RTVD4bl6	...	...	...	...	...	...	...	...	...	...	..G	...	.G.	...	...	...	...	...	...	...	...	...	...	...	...	...
RTVD4bl21	...	...	...	...	...	...	...	...	...	...	..G	...	.G.	...	...	...	...	...	...	...	...	C..	..C	...	...	...
RTVD4bl2	...	...	...	...	...	...	...	...	...	...	..G	...	.G.	...	...	...	...	...	...	...	...	...	...	...	...	...
RTVD4bl32	...	...	...	...	...	...	...	...	...	...	...	...	...	...	.A.	...	...	...	...	...	...	...	...	...	...	..G
RTVD4bl10	...	...	...	...	...	.C.	.G.	...	...	...	...	...	...	...	.A.	...	...	...	...	...	...	...	...	...	...	..G
RTVD4bl15	...	...	...	...	...	.C.	.G.	...	...	...	...	...	...	...	.A.	...	...	...	...	...	...	...	...	...	...	..G
RTVD4bl31	...	...	...	...	...	.C.	.G.	...	...	...	...	...	...	...	.A.	...	...	...	...	...	...	...	...	...	...	..G

	122	124	125	126	127	128	Total Mutations	Tandem Groups
	T	L	N	V	E	P		
VD4.14	ACC	CTG	AAT	GTT	GAA	CCA		
RTVD4m14	...	...	...	...	.C.	...	11	1
RTVD4m17	...	...	...	...	.C.	...	6	1
RTVD4m18	...	...	...	...	.C.	...	3	1
RTVD4m29	...	...	...	...	.C.	...	3	0
RTVD4m30	...	...	...	.C.	...	...	6	1
RTVD4m16	...	...	..A	...	...	...	6	0
RTVD4m5	...	...	...	...	...	...	8	0
RTVD4m26	...	G..	...	...	..G	...	6	0
RTVD4m10	...	...	...	...	...	...	2	0
RTVD4m25	...	...	...	...	...	...	5	0
RTVD4m4	...	...	...	...	...	...	2	0
SC43	...	...	...	...	...	...	2	0
RTVD4m9	...	...	...	...	...	..G	3	0
							63*	4*
RTVD4bl7	...	...	..A	...	...	...	4	
RTVD4bl6	...	...	..A	...	...	...	4	
RTVD4bl21	...	...	..A	...	...	...	4	
RTVD4bl2	...	...	..A	...	...	...	1	
RTVD4bl32	..G	.G.	.T.	...	...	...	5	
RTVD4bl10	...	...	...	...	...	...	4	
RTVD4bl15	...	...	...	...	...	...	4	
RTVD4bl31	...	...	...	...	...	...	1	
							27*	

Colours	Related* Mutations	
Green	Transition	C or T
Blue	Transition	A or G
Bordeaux	Transversion	G or C
Pink	Transversion	A or C
Orange	Transversion	G or T
Red	Transversion	A or T
