# Supplementary material for: 3D structures inferred from cDNA clones identify the CD1D-Restricted γδ T cell receptor in dromedaries
Source: Front Immunol. 2022 Aug 9;13:928860. doi: 10.3389/fimmu.2022.928860 (PMC9396240; doi:10.3389/fimmu.2022.928860)
Supplement: Supplementary file 6 [file Image_5.pdf]

A

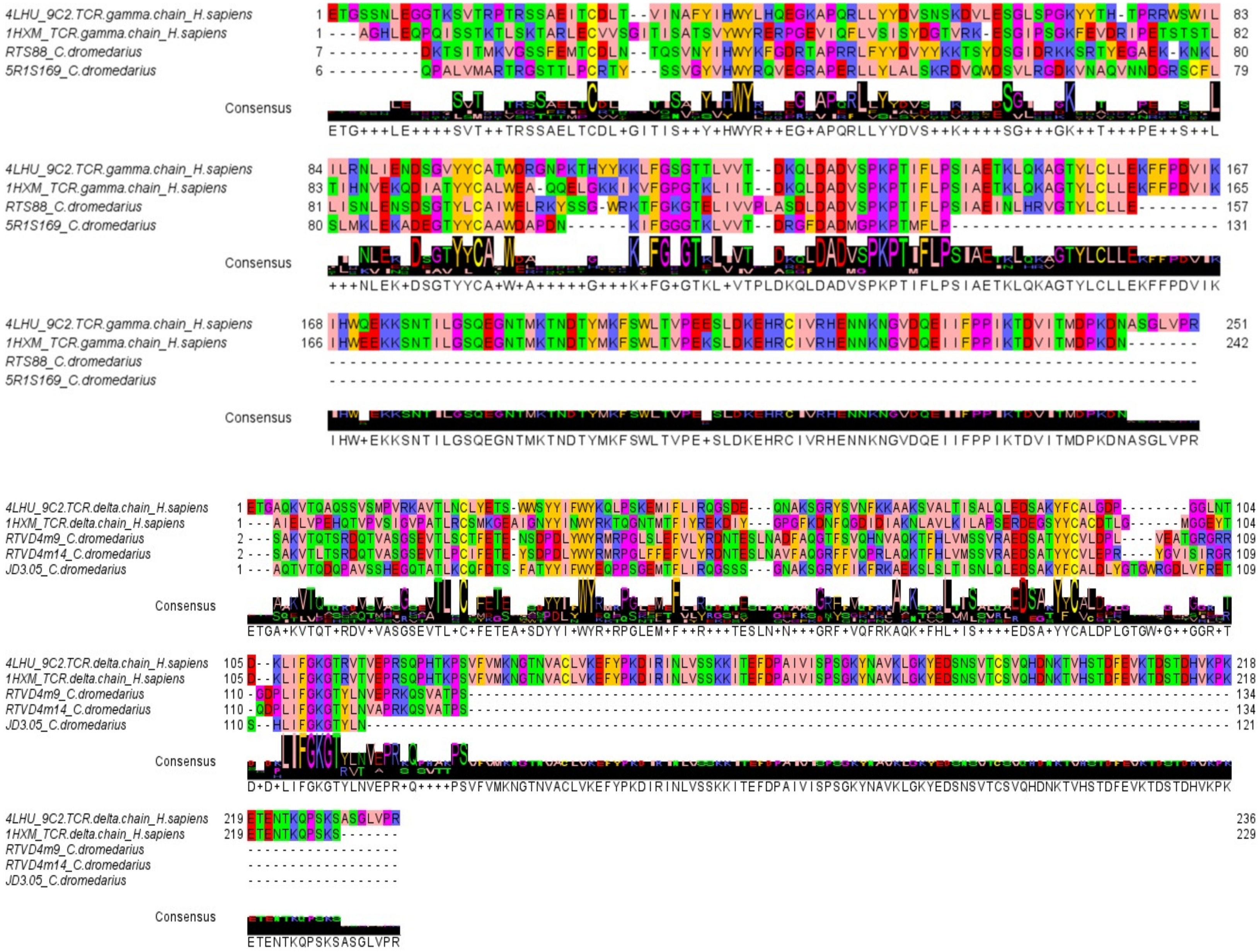

Sequence-structure alignment of the investigated *C. dromedarius* TRG and TRD chains with human TRG and TRD chains from the crystallized structures 4lhu.pdb
