# Supplementary material for: 3D structures inferred from cDNA clones identify the CD1D-Restricted γδ T cell receptor in dromedaries
Source: Front Immunol. 2022 Aug 9;13:928860. doi: 10.3389/fimmu.2022.928860 (PMC9396240; doi:10.3389/fimmu.2022.928860)
Supplement: Supplementary file 7 [file Image_6.pdf]

B

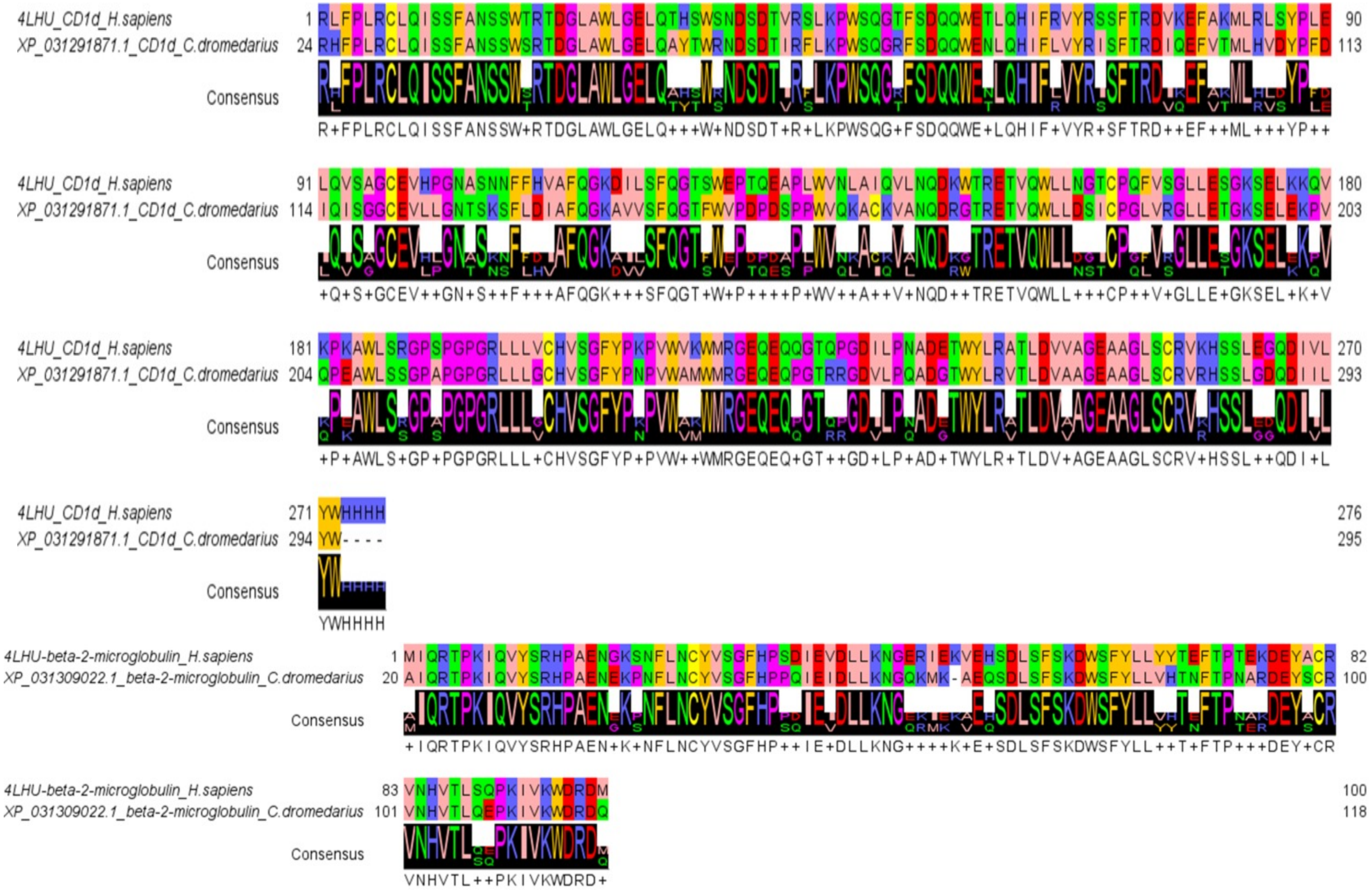

Sequence-structure pairwise alignment of the human CD1D (from 4lhu.pdb) and beta-2-microglobulin (from 4lhu.pdb) with their closest homologues in *C. dromedarius*
