# Supplementary material for: 3D structures inferred from cDNA clones identify the CD1D-Restricted γδ T cell receptor in dromedaries
Source: Front Immunol. 2022 Aug 9;13:928860. doi: 10.3389/fimmu.2022.928860 (PMC9396240; doi:10.3389/fimmu.2022.928860)
Supplement: Supplementary file 8 [file Image_7.pdf]

RTS88_RTVD4m9

1) Protein-protein Ionic Interactions

1.1 Ionic interactions within 6 Angstroms

Chain	Position	Residue	FR/CDR	Chain	Position	Residue	FR/CDR
TRG	52	ARG (R)	FR2	TRD	111	GLU (E)	CDR3
TRG	57	ASP (D)	CDR2	TRD	112.1	ARG (R)	CDR3
TRG	116	LYS (K)	CDR3	TRD	107	ASP (D)	CDR3
TRG	116	LYS (K)	CDR3	TRD	114	ASP (D)	CDR3

Chain	Position	Residue	FR/CDR	Chain	Position	Residue	G-ALPHA-LIKE
TRG	64	LYS (K)	CDR2	RPI MH1 LIKE	43	ASP (D)	G-ALPHA1
TRD	112.1	ARG (R)	CDR3	RPI MH1 LIKE	61	GLU (E)	G-ALPHA1
TRD	112	ARG (R)	CDR3	RPI MH1 LIKE	61	GLU (E)	G-ALPHA1
TRD	56	ARG (R)	CDR2	RPI MH1 LIKE	65	GLU (E)	G-ALPHA2

2) Protein-protein Side Chain-Side Chain Hydrogen Bonds

Chain	Position	Residue	FR/CDR	Chain	Position	Residue	FR/CDR
TRG	38	TYR (Y)	CDR1	TRD	112.1	ARG (R)	CDR3
TRG	55	TYR (Y)	FR2	TRD	112.1	ARG (R)	CDR3
TRG	57	ASP (D)	CDR2	TRD	112.1	ARG (R)	CDR3
TRG	111.1	TYR (Y)	CDR3	TRD	72	GLN (Q)	FR3
TRG	114	TRP (W)	CDR3	TRD	38	ASP (D)	CDR1
TRG	116	LYS (K)	CDR3	TRD	107	ASP (D)	CDR3

Chain	Position	Residue	FR/CDR	Chain	Position	Residue	G-ALPHA-LIKE
TRG	38	TYR (Y)	CDR1	RPI MH1 LIKE	65	HIS (H)	G-ALPHA1
TRG	52	ARG (R)	FR2	RPI MH1 LIKE	58	GLN (Q)	G-ALPHA1
TRG	56	TYR (Y)	CDR2	RPI MH1 LIKE	61	GLU (E)	G-ALPHA1
TRD	29	ASN (N)	CDR1	RPI MH1 LIKE	59	GLN (Q)	G-ALPHA1
TRD	112.1	ARG (R)	CDR3	RPI MH1 LIKE	61	GLU (E)	G-ALPHA1
TRD	112	ARG (R)	CDR3	RPI MH1 LIKE	58	GLN (Q)	G-ALPHA1
TRD	112	ARG (R)	CDR3	RPI MH1 LIKE	61	GLU (E)	G-ALPHA1
TRD	112	ARG (R)	CDR3	RPI MH1 LIKE	62	ASN (N)	G-ALPHA1

3) Protein-Protein Aromatic-Aromatic Interactions

3.1 Aromatic-Aromatic Interactions within 4.5 and 7 Angstrom

Chain	Position	Residue	FR/CDR	Chain	Position	Residue	FR/CDR
TRG	42	TYR (Y)	FR2	TRD	118	PHE (F)	CDR3
TRG	44	PHE (F)	FR2	TRD	103	TYR (Y)	FR3
TRG	111.1	TYR (Y)	CDR3	TRD	52	PHE (F)	FR2
TRG	118	PHE (F)	CDR3	TRD	118	PHE (F)	CDR3
TRG	118	PHE (F)	CDR3	TRD	42	TYR (Y)	FR2

4) Protein-Protein Aromatic-Sulphur Interactions

Chain	Position	Residue	FR/CDR	Chain	Position	Residue	FR/CDR
TRG	44	PHE (F)	FR2	TRD	44	MET (M)	FR2

5) Protein-Protein Cation-Pi Interactions

Chain	Position	Residue	FR/CDR	Chain	Position	Residue	FR/CDR
TRG	38	TYR (Y)	CDR1	TRD	112.1	ARG (R)	CDR3
TRG	56	TYR (Y)	CDR2	TRD	112.1	ARG (R)	CDR3
TRG	56	TYR (Y)	CDR2	TRD	112	ARG (R)	CDR3
TRG	115	ARG (R)	CDR3	TRD	52	PHE (F)	FR2

6) Protein-Protein Hydrophobic Interactions

6.1 Hydrophobic Interactions within 5 Angstroms

Chain	Position	Residue	FR/CDR	Chain	Position	Residue	FR/CDR
TRG	42	TYR (Y)	FR2	TRD	115	PRO (P)	CDR3
TRG	42	TYR (Y)	FR2	TRD	116	LEU (L)	CDR3
TRG	42	TYR (Y)	FR2	TRD	118	PHE (F)	CDR3
TRG	44	PHE (F)	FR2	TRD	44	MET (M)	FR2
TRG	44	PHE (F)	FR2	TRD	48	LEU (L)	FR2
TRG	44	PHE (F)	FR2	TRD	103	TYR (Y)	FR3
TRG	49	ALA (A)	FR2	TRD	118	PHE (F)	CDR3
TRG	50	PRO (P)	FR2	TRD	118	PHE (F)	CDR3
TRG	50	PRO (P)	FR2	TRD	44	MET (M)	FR2
TRG	50	PRO (P)	FR2	TRD	103	TYR (Y)	FR3
TRG	103	LEU (L)	FR3	TRD	50	LEU (L)	FR2
TRG	111.1	TYR (Y)	CDR3	TRD	52	PHE (F)	FR2
TRG	118	PHE (F)	CDR3	TRD	116	LEU (L)	CDR3
TRG	118	PHE (F)	CDR3	TRD	118	PHE (F)	CDR3
TRG	118	PHE (F)	CDR3	TRD	42	TYR (Y)	FR2
TRG	118	PHE (F)	CDR3	TRD	50	LEU (L)	FR2

Chain	Position	Residue	FR/CDR	Chain	Position	Residue	G-ALPHA-LIKE
TRD	111.1	ALA (A)	CDR3	RPI MH1 LIKE	69	TRP (W)	G-ALPHA2
