# Supplementary material for: 3D structures inferred from cDNA clones identify the CD1D-Restricted γδ T cell receptor in dromedaries
Source: Front Immunol. 2022 Aug 9;13:928860. doi: 10.3389/fimmu.2022.928860 (PMC9396240; doi:10.3389/fimmu.2022.928860)
Supplement: Supplementary file 9 [file Image_8.pdf]

**RTS88_RTVD4m14**

1) Protein-protein Ionic Interactions
   1.1 Ionic interactions within 6 Angstroms

| Chain | Position | Residue | FR/CDR | Chain | Position | Residue | FR/CDR |
|---|---|---|---|---|---|---|---|
| TRG | 52 | ARG (R) | FR2 | TRD | 114 | ASP (D) | CDR3 |
| TRG | 68 | ASP (D) | FR3 | TRD | 112 | ARG (R) | CDR3 |
| TRG | 116 | LYS (K) | CDR3 | TRD | 107 | GLU (E) | CDR3 |

| Chain | Position | Residue | FR/CDR | Chain | Position | Residue | G-ALPHA-LIKE |
|---|---|---|---|---|---|---|---|
| TRG | 57 | ASP (D) | CDR2 | RPI MH1 LIKE | 65 | HIS (H) | G-ALPHA1 |
| TRD | 112 | ARG (R) | CDR3 | RPI MH1 LIKE | 61 | GLU (E) | G-ALPHA1 |

2) Protein-protein Side Chain-Side Chain Hydrogen Bonds

| Chain | Position | Residue | FR/CDR | Chain | Position | Residue | FR/CDR |
|---|---|---|---|---|---|---|---|
| TRG | 40 | HIS (H) | FR2 | TRD | 113 | GLN (Q) | CDR3 |
| TRG | 115 | ARG (R) | CDR3 | TRD | 40 | TYR (Y) | FR2 |
| TRG | 116 | LYS (K) | CDR3 | TRD | 107 | GLU (E) | CDR3 |

| Chain | Position | Residue | FR/CDR | Chain | Position | Residue | G-ALPHA-LIKE |
|---|---|---|---|---|---|---|---|
| TRG | 38 | TYR (Y) | CDR1 | RPI MH1 LIKE | 65 | HIS (H) | G-ALPHA1 |
| TRG | 55 | TYR (Y) | CDR2 | RPI MH1 LIKE | 61 | GLU (E) | G-ALPHA1 |
| TRG | 68 | ASP (D) | FR3 | RPI MH1 LIKE | 58 | GLN (Q) | G-ALPHA1 |
| TRD | 36 | ASP (D) | CDR1 | RPI MH1 LIKE | 72A | SER (S) | G-ALPHA2 |
| TRD | 58 | ASN (N) | CDR2 | RPI MH1 LIKE | 69 | TRP (W) | G-ALPHA2 |
| TRD | 111.3 | SER (S) | CDR3 | RPI MH1 LIKE | 62 | ASN (N) | G-ALPHA1 |
| TRD | 112 | ARG (R) | CDR3 | RPI MH1 LIKE | 58 | GLN (Q) | G-ALPHA1 |

3) Protein-Protein Aromatic-Aromatic Interactions

3.1 Aromatic-Aromatic Interactions within 4.5 and 7 Angstrom

| Chain | Position | Residue | FR/CDR | Chain | Position | Residue | FR/CDR |
|---|---|---|---|---|---|---|---|
| TRG | 42 | TYR (Y) | FR2 | TRD | 118 | PHE (F) | CDR3 |
| TRG | 42 | TYR (Y) | FR2 | TRD | 50 | PHE (F) | FR2 |
| TRG | 44 | PHE (F) | FR2 | TRD | 103 | TYR (Y) | FR3 |
| TRG | 111.1 | TYR (Y) | CDR3 | TRD | 52 | PHE (F) | FR2 |
| TRG | 114 | TRP (W) | CDR3 | TRD | 110 | TYR (Y) | CDR3 |
| TRG | 118 | PHE (F) | CDR3 | TRD | 118 | PHE (F) | CDR3 |
| TRG | 118 | PHE (F) | CDR3 | TRD | 42 | TYR (Y) | FR2 |
| TRG | 118 | PHE (F) | CDR3 | TRD | 50 | PHE (F) | FR2 |

| Chain | Position | Residue | FR/CDR | Chain | Position | Residue | G-ALPHA-LIKE |
|---|---|---|---|---|---|---|---|
| TRG | 114 | TRP (W) | CDR3 | RPI MH1 LIKE | 69 | TRP (W) | G-ALPHA2 |
| TRD | 29 | TYR (Y) | CDR1 | RPI MH1 LIKE | 55 | PHE (F) | G-ALPHA1 |
| TRD | 110 | TYR (Y) | CDR3 | RPI MH1 LIKE | 69 | TRP (W) | G-ALPHA2 |

## 4) Protein-Protein Aromatic-Sulphur Interactions

| Chain | Position | Residue | FR/CDR | Chain | Position | Residue | FR/CDR |
|---|---|---|---|---|---|---|---|
| TRG | 44 | PHE (F) | FR2 | TRD | 44 | MET (M) | FR2 |

## 5) Protein-Protein Cation-Pi Interactions

| Chain | Position | Residue | FR/CDR | Chain | Position | Residue | FR/CDR |
|---|---|---|---|---|---|---|---|
| TRG | 55 | TYR (Y) | CDR2 | TRD | 112 | ARG (R) | CDR3 |
| TRG | 110 | ARG (R) | CDR3 | TRD | 52 | PHE (F) | FR2 |
| TRG | 114 | TRP (W) | CDR3 | TRD | 56 | ARG (R) | CDR2 |
| TRG | 115 | ARG (R) | CDR3 | TRD | 40 | TYR (Y) | FR2 |
| TRG | 115 | ARG (R) | CDR3 | TRD | 52 | PHE (F) | FR2 |
| TRG | 116 | LYS (K) | CDR3 | TRD | 40 | TYR (Y) | FR2 |
| TRG | 116 | LYS (K) | CDR3 | TRD | 110 | TYR (Y) | CDR3 |
| TRG | 120 | LYS (K) | FR4 | TRD | 49 | PHE (F) | FR2 |

| Chain | Position | Residue | FR/CDR | Chain | Position | Residue | G-ALPHA-LIKE |
|---|---|---|---|---|---|---|---|
| TRD | 109 | ARG (R) | CDR3 | RPI-MH1Like | 69 | TRP (W) | G-ALPHA2 |

## 6) Protein-Protein Hydrophobic Interactions

### 6.1 Hydrophobic Interactions within 5 Angstroms

| Chain | Position | Residue | FR/CDR | Chain | Position | Residue | FR/CDR |
|---|---|---|---|---|---|---|---|
| TRG | 38 | TYR (Y) | CDR1 | TRD | 111.2 | ILE (I) | CDR3 |
| TRG | 42 | TYR (Y) | FR2 | TRD | 118 | PHE (F) | CDR3 |
| TRG | 42 | TYR (Y) | FR2 | TRD | 50 | PHE (F) | FR2 |
| TRG | 44 | PHE (F) | FR2 | TRD | 44 | MET (M) | FR2 |
| TRG | 44 | PHE (F) | FR2 | TRD | 48 | LEU (L) | FR2 |
| TRG | 44 | PHE (F) | FR2 | TRD | 103 | TYR (Y) | FR3 |
| TRG | 49 | ALA (A) | FR2 | TRD | 118 | PHE (F) | CDR3 |
| TRG | 50 | PRO (P) | FR2 | TRD | 118 | PHE (F) | CDR3 |
| TRG | 50 | PRO (P) | FR2 | TRD | 50 | PHE (F) | FR2 |
| TRG | 50 | PRO (P) | FR2 | TRD | 103 | TYR (Y) | FR3 |
| TRG | 103 | LEU (L) | FR3 | TRD | 50 | PHE (F) | FR2 |
| TRG | 107 | TRP (W) | CDR3 | TRD | 111.2 | ILE (I) | CDR3 |
| TRG | 111.1 | TYR (Y) | CDR3 | TRD | 52 | PHE (F) | FR2 |

| | | | | | | | |
|---|---|---|---|---|---|---|---|
| TRG | 114 | TRP (W) | CDR3 | TRD | 110 | TYR (Y) | CDR3 |
| TRG | 118 | PHE (F) | CDR3 | TRD | 116 | LEU (L) | CDR3 |
| TRG | 118 | PHE (F) | CDR3 | TRD | 118 | PHE (F) | CDR3 |
| TRG | 118 | PHE (F) | CDR3 | TRD | 42 | TYR (Y) | FR2 |
| TRG | 118 | PHE (F) | CDR3 | TRD | 50 | PHE (F) | FR2 |

| Chain | Position | Residue | FR/CDR | Chain | Position | Residue | G-ALPHA-LIKE |
|---|---|---|---|---|---|---|---|
| TRG | 62 | TYR (Y) | CDR2 | RPI MH1 LIKE | 68 | LEU (L) | G-ALPHA1 |
| TRG | 114 | TRP (W) | CDR3 | RPI MH1 LIKE | 69 | TRP (W) | G-ALPHA2 |
| TRD | 29 | TYR (Y) | CDR1 | RPI MH1 LIKE | 55 | PHE (F) | G-ALPHA1 |
| TRD | 29 | TYR (Y) | CDR1 | RPI MH1 LIKE | 73 | ILE (I) | G-ALPHA2 |
| TRD | 110 | TYR (Y) | CDR3 | RPI MH1 LIKE | 69 | TRP (W) | G-ALPHA2 |
| TRD | 111.1 | VAL (V) | CDR3 | RPI MH1 LIKE | 66 | ILE (I) | G-ALPHA1 |
