# Supplementary material for: 3D structures inferred from cDNA clones identify the CD1D-Restricted γδ T cell receptor in dromedaries
Source: Front Immunol. 2022 Aug 9;13:928860. doi: 10.3389/fimmu.2022.928860 (PMC9396240; doi:10.3389/fimmu.2022.928860)
Supplement: Supplementary file 10 [file Image_9.pdf]

5R1S169_JD3.05

1) Protein-protein Ionic Interactions

1.1 Ionic interactions within 6 Angstroms

Chain	Position	Residue	FR/CDR	Chain	Position	Residue	FR/CDR
TRG	46	GLU (E)	FR2	TRD	101	LYS (K)	FR3
TRG	52	ARG (R)	FR2	TRD	112	GLU (E)	CDR3
TRG	116	LYS (K)	CDR3	TRD	107	ASP (D)	CDR3

Chain	Position	Residue	FR/CDR	Chain	Position	Residue	G-ALPHA-LIKE
TRD	55	ARG (R)	CDR2	RPI MH1 LIKE	65	GLU (E)	G-ALPHA2

2) Protein-protein Side Chain-Side Chain Hydrogen Bonds

Chain	Position	Residue	FR/CDR	Chain	Position	Residue	FR/CDR
TRG	44	GLN (Q)	FR2	TRD	44	GLN (Q)	FR2
TRG	44	GLN (Q)	FR2	TRD	50	MET (M)	FR2
TRG	52	ARG (R)	FR2	TRD	112	GLU (E)	CDR3
TRG	52	ARG (R)	FR2	TRD	115	HIS (H)	CDR3
TRG	116	LYS (K)	CDR3	TRD	107	ASP (D)	CDR3

Chain	Position	Residue	FR/CDR	Chain	Position	Residue	G-ALPHA-LIKE
TRG	38	TYR (Y)	CDR1	RPI MH1 LIKE	65	HIS (H)	G-ALPHA1
TRD	36	THR (T)	CDR1	RPI MH1 LIKE	72A	SER (S)	G-ALPHA2
TRD	38	TYR (Y)	CDR1	RPI MH1 LIKE	65	GLU (E)	G-ALPHA2
TRD	109	TYR (Y)	CDR3	RPI MH1 LIKE	59	GLN (Q)	G-ALPHA1
TRD	112.5	ASP (D)	CDR3	RPI MH1 LIKE	62	ASN (N)	G-ALPHA1
TRD	113	THR (T)	CDR3	RPI MH1 LIKE	58	GLN (Q)	G-ALPHA1

3) Protein-Protein Aromatic-Aromatic Interactions

3.1 Aromatic-Aromatic Interactions within 4.5 and 7 Angstrom

Chain	Position	Residue	FR/CDR	Chain	Position	Residue	FR/CDR
TRG	42	TYR (Y)	FR2	TRD	118	PHE (F)	CDR3
TRG	118	PHE (F)	CDR3	TRD	118	PHE (F)	CDR3
TRG	118	PHE (F)	CDR3	TRD	42	TYR (Y)	FR2

Chain	Position	Residue	FR/CDR	Chain	Position	Residue	G-ALPHA-LIKE
TRD	29	PHE (F)	CDR1	RPI MH1 LIKE	55	PHE (F)	G-ALPHA1
TRD	38	(TYR) (Y)	CDR1	RPI MH1 LIKE	69	TRP (W)	G-ALPHA2
TRD	109	(TYR) (Y)	CDR3	RPI MH1 LIKE	55	PHE (F)	G-ALPHA1

4) Protein-Protein Aromatic-Sulphur Interactions

Chain	Position	Residue	FR/CDR	Chain	Position	Residue	FR/CDR
TRG	42	(TYR) (Y)	FR2	TRD	50	MET (M)	FR2
TRG	103	(TYR) (Y)	FR3	TRD	50	MET (M)	FR2
TRG	118	PHE (F)	CDR3	TRD	50	MET (M)	FR2

5) Protein-Protein Cation-Pi Interactions

Chain	Position	Residue	FR/CDR	Chain	Position	Residue	FR/CDR
TRG	42	(TYR) (Y)	FR2	TRD	112.1	ARG (R)	CDR3
TRG	116	(LYS) (K)	CDR3	TRD	40	PHE (F)	FR2
TRG	116	(LYS) (K)	CDR3	TRD	111.2	TRP (W)	CDR3
TRG	118	PHE (F)	CDR3	TRD	112.1	ARG (R)	CDR3

6) Protein-Protein Hydrophobic Interactions

6.1 Hydrophobic Interactions within 5 Angstroms

Chain	Position	Residue	FR/CDR	Chain	Position	Residue	FR/CDR
TRG	38	TYR (Y)	CDR1	TRD	112.4	LEU (L)	CDR3
TRG	38	TYR (Y)	CDR1	TRD	112.3	VAL (V)	CDR3
TRG	42	TYR (Y)	FR2	TRD	118	PHE (F)	CDR3
TRG	42	TYR (Y)	FR2	TRD	50	MET (M)	FR2
TRG	49	ALA (A)	FR2	TRD	118	PHE (F)	CDR3
TRG	50	PRO (P)	FR2	TRD	118	PHE (F)	CDR3
TRG	50	PRO (P)	FR2	TRD	103	PHE (F)	FR3
TRG	55	TYR (Y)	CDR2	TRD	112.3	VAL (V)	CDR3
TRG	103	TYR (Y)	FR3	TRD	50	MET (M)	FR2
TRG	107	TRP (W)	CDR3	TRD	111.2	TRP (W)	CDR3
TRG	107	TRP (W)	CDR3	TRD	112.4	LEU (L)	CDR3
TRG	117	ILE (I)	CDR3	TRD	52	PHE (F)	FR2
TRG	118	PHE (F)	CDR3	TRD	116	LEU (L)	CDR3
TRG	118	PHE (F)	CDR3	TRD	118	PHE (F)	CDR3
TRG	118	PHE (F)	CDR3	TRD	42	TYR (Y)	FR2
TRG	118	PHE (F)	CDR3	TRD	50	MET (M)	FR2

Chain	Position	Residue	FR/CDR	Chain	Position	Residue	G-ALPHA-LIKE
TRD	29	PHE (F)	CDR1	RPI MH1 LIKE	69	TRP (W)	G-ALPHA2
TRD	29	PHE (F)	CDR1	RPI MH1 LIKE	73	ILE (I)	G-ALPHA2
TRD	29	PHE (F)	CDR1	RPI MH1 LIKE	55	PHE (F)	G-ALPHA1
TRD	38	TYR (Y)	CDR1	RPI MH1 LIKE	69	TRP (W)	G-ALPHA2
TRD	109	TYR (Y)	CDR3	RPI MH1 LIKE	69	TRP (W)	G-ALPHA2
TRD	109	TYR (Y)	CDR3	RPI MH1 LIKE	73	ILE (I)	G-ALPHA2
TRD	109	TYR (Y)	CDR3	RPI MH1 LIKE	55	PHE (F)	G-ALPHA1
TRD	109	TYR (Y)	CDR3	RPI MH1 LIKE	63	LEU (L)	G-ALPHA1
TRD	109	TYR (Y)	CDR3	RPI MH1 LIKE	66	ILE (I)	G-ALPHA1
